# *J* Coupling Constants of <1 Hz Enable ^13^C Hyperpolarization of Pyruvate via Reversible Exchange of Parahydrogen

**DOI:** 10.1021/acs.jpclett.3c02980

**Published:** 2024-01-25

**Authors:** Charbel D. Assaf, Xin Gui, Alexander A. Auer, Simon B. Duckett, Jan-Bernd Hövener, Andrey N. Pravdivtsev

**Affiliations:** † Section Biomedical Imaging, Molecular Imaging North Competence Center (MOIN CC), Department of Radiology and Neuroradiology, University Medical Center Kiel, Kiel University, Am Botanischen Garten 14, 24118 Kiel, Germany; ‡ Max-Planck-Institut für Kohlenforschung, Kaiser-Wilhelm-Platz 1, 45470 Mülheim an der Ruhr, Germany; § Centre for Hyperpolarization in Magnetic Resonance (CHyM), University of York, Heslington YO10 5NY, U.K.

## Abstract

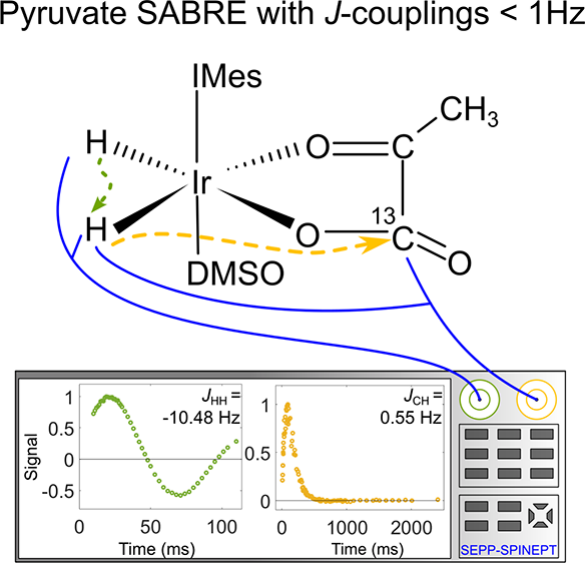

Observing pyruvate metabolism *in vivo* has become a focal point of molecular magnetic resonance imaging. Signal amplification by reversible exchange (SABRE) has recently emerged as a versatile hyperpolarization technique. Tuning of the spin order transfer (SOT) in SABRE is challenging as the small ^1^H–^13^C *J* couplings, in the ^13^C-pyruvate case, result in SOT being not readily discernible. We demonstrate an experimental method using frequency-selective excitation of parahydrogen-derived polarization SOT sequence (SEPP-SPINEPT); its application led to up to 5700-fold ^13^C signal gain. In this way, we estimated the lifetime of two Ir–pyruvate SABRE complexes alongside the individual probing of eight small ^1^H–^13^C *J* couplings that connect the hydride protons in these complexes to 1- and 2-^13^C pyruvate spins, affording values between 0 and 2.69 Hz. Using electronic structure calculations, we define the low-energy structure of the corresponding complexes. Hence, this study demonstrates a novel approach to analyzing the spin topology of short-lived organometallic complexes.

Pyruvate (pyr) plays a significant role in cellular metabolism, lying at the junction of numerous metabolic processes within living cells.^1−3^ Studying the metabolic fate of pyr has attracted a great deal of attention due to its potential as a clinical tool, particularly in diagnosing cancer^4^ and inflammation.^5^ Nuclear magnetic resonance (NMR) and magnetic resonance imaging (MRI) allow the tracking of pyr metabolism *in vitro* and *in vivo*. However, these methods are insensitive to the low-spin polarization produced by the magnetic fields available today. Several techniques can increase nuclear spin polarization, known as hyperpolarization; pyr was hyperpolarized using dissolution dynamic nuclear polarization (dDNP)^4,6,7^ more than a decade ago. Later, parahydrogen-induced polarization with side arm hydrogenation (PHIP-SAH)^8−13^ was used to polarize pyr, and only recently, was this achieved by signal amplification by reversible exchange (SABRE).^14−17^

SABRE^14^ is particularly interesting because it is fast and allows continuous hyperpolarization.^18−20^ In contrast to PHIP-SAH, in which an unsaturated precursor is required,^8−13^ a chemical modification of the agent is unnecessary. These attributes make SABRE more accessible than dDNP and PHIP-SAH, leading to a rapid *in vivo* demonstration.^21,22^

In SABRE, the target substrate (here pyr) and parahydrogen (pH_2_) coordinate reversibly to an Ir complex (Figure 1) so that a transient *J* coupling is created between pyr and protons previously in pH_2_. These couplings allow the transfer of spin alignment to pyruvate,^14,15^ which can be used later for imaging or spectroscopy. We will refer to the two protons of pH_2_, now coordinated to the Ir complex, as IrHH. There are two main mechanisms for coherent SOT in SABRE: free evolution at (low) magnetic fields, which takes place close to level anticrossings of the involved spin states,^23−27^ or free evolution in combination with radiofrequency (RF) excitation at a low or high magnetic field.^28−32^ Polarization transfer via incoherent interactions has been demonstrated, too, but these mechanisms are usually less efficient.^33−35^

**Figure 1:**
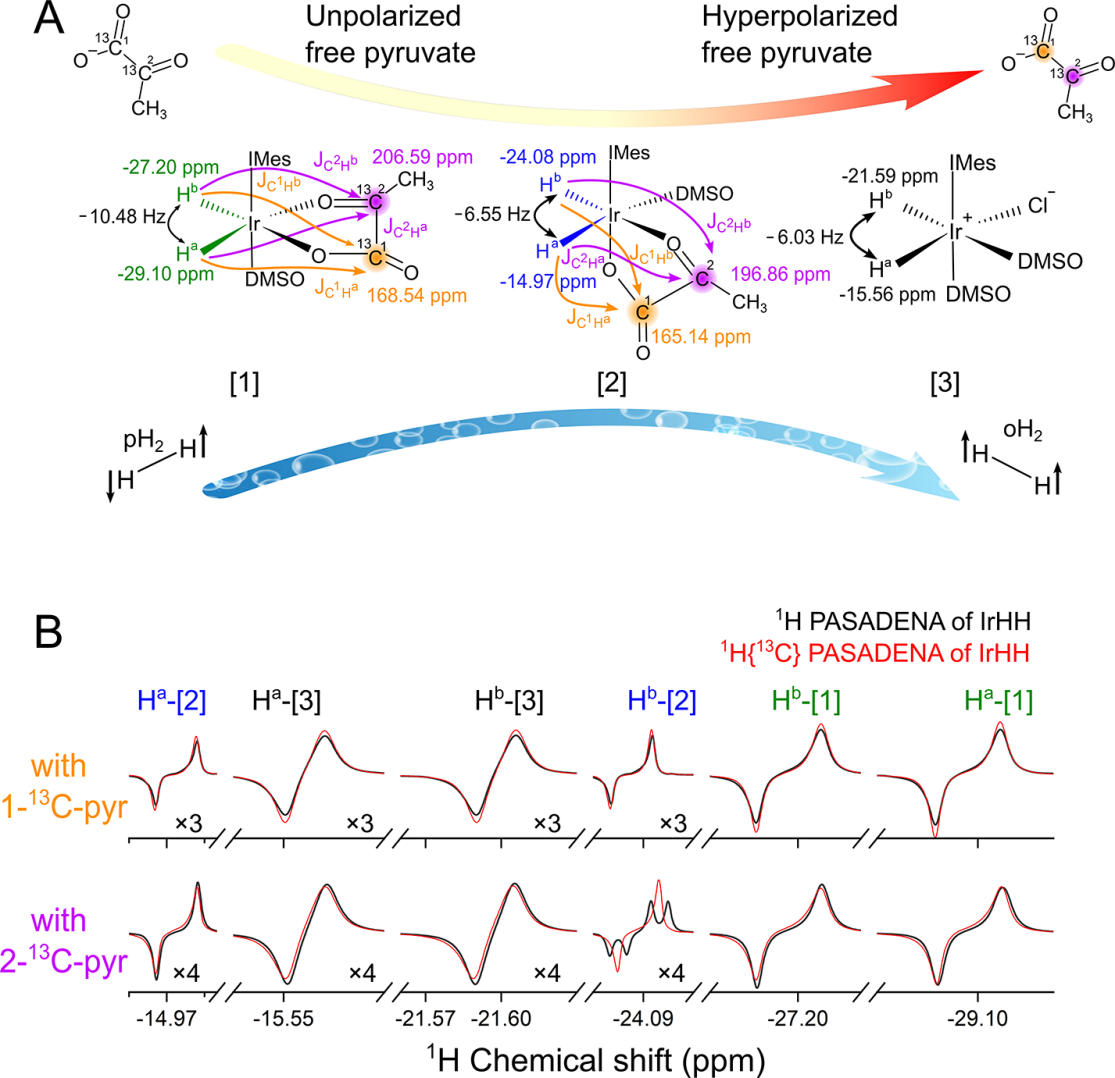
SABRE hyperpolarization of ^13^C-pyr and IrHH protons. (A) H_2_, DMSO, and pyr bind to the transient, active, Ir-based SABRE complex [Ir(H)_2_(pyr)(DMSO)(IMes)] for [**1**] and [**2**], and [Ir(H)_2_(Cl)(DMSO)_2_(IMes)] for [**3**]. *J* couplings under appropriate conditions transfer polarization from IrHH (pH_2_-derived hydride protons) to pyr. There are three primary hyperpolarized complexes, [**1**]–[**3**]: two where pyr binds on equatorial, [**1**], or axial–equatorial, [**2**], sites and a third complex with an additional DMSO ligand, [**3**]. (B) ^1^H (black) and ^1^H{^13^C} (red) NMR PASADENA spectra showing polarized IrHH protons of [**1**]–[**3**] formed by supplying 8.75 bar of pH_2_ to the Ir catalyst precursor with either 1-^13^C-pyr (top) or 2-^13^C-pyr (bottom) in methanol-*d*_4_ at 267 K. The spectra consist of three pairs of hydride signals, with the dominant signal being from [**1**]. The ^1^H chemical shift was calibrated to CD_2_HOD to be 3.34 ppm.^36^

In the presence of H_2_, dimethyl sulfoxide (DMSO), pyr (CH_3_COCO_2_^−^, added as the sodium salt of pyruvate), and an IrIMes catalyst precursor {[IrCl(COD)(IMes)], where COD = cyclooctadiene, and IMes = 1,3-bis(2,4,6-trimethylphenyl)imidazol-2-ylidene}, three major complexes result: [Ir(H)_2_(pyr)(DMSO)(IMes)] complexes [**1**] and **[2]**, and [Ir(H)_2_(Cl)(DMSO)_2_(IMes)] complex [**3**] (Figure 1). Under pH_2_ pressure at low temperatures, the mechanism of H_2_ exchange operates by opening the chelate in [**1**], which makes the pyruvate ligand η-1.^15^ New pH_2_ can then exchange with the already bound IrHH protons, and the chelate re-forms, resulting in the hyperpolarized signal for complex [**1**] (Figure 1B). A similar mechanism takes place for [**2**] and [**3**]. Additionally, DMSO ligand exchanges are most likely to occur in [**2**] and [**3**] rather than in [**1**], as the hydride is a good *trans*-labilizing ligand.

So far, the highest pH_2_-based polarization of 1-^13^C-pyr (>20%) with SABRE was achieved at 50 μT using weak RF irradiation and fully deuterated pyr.^32^ These impressive polarization levels highlight the potential of SABRE to enhance the sensitivity and detection of pyr for a wide range of applications.

Interestingly, *J* couplings mediate the polarization transfer in all coherence-driven SOTs. To optimize the SOT conditions, one must know the size of all such interactions. Unfortunately, these parameters are unknown for the SABRE active catalysts, Ir–pyr complexes [**1**] and [**2**] (Figure 1). This is due to the inability of ^1^H NMR to detect these couplings because of their small magnitude and line broadening due to chemical exchange. In recent work,^27^ these parameters were estimated using density functional theory (DFT) simulations and SOT at low magnetic fields; however, this approach has known biases.

To assess the details of the molecular structures of the complexes investigated, we carried out electronic structure calculations at the DFT level of theory. For [**1**]–[**3**] (Figure S14), geometry optimizations and normal-mode analyses were carried out for a series of possible conformers.

The relative Gibbs free energy of compound [**2**] (Figure 2D) is higher than that of complex [**1**] by 7.6 kJ/mol. In addition, the relative Gibbs free energy of isomer [**2′**] (Figure 2E),^15^ with the pyr coordinated differently than in [**2**], is 11.9 kJ/mol higher than that of [**2**] (and 19.5 kJ/mol higher than that of [**1**]), indicating that experimentally one likely observes [**2**]^37^ instead of [**2′**].^15^ Similarly, for [**3**], there is an isomer, [**3′**], with an energy difference of 0.4 kJ/mol, which differs by rotation of the IMes structure (Figure S14C). Hence, we will assign the observed experimental interactions to primary isomers [**1**]–[**3**].

**Figure 2:**
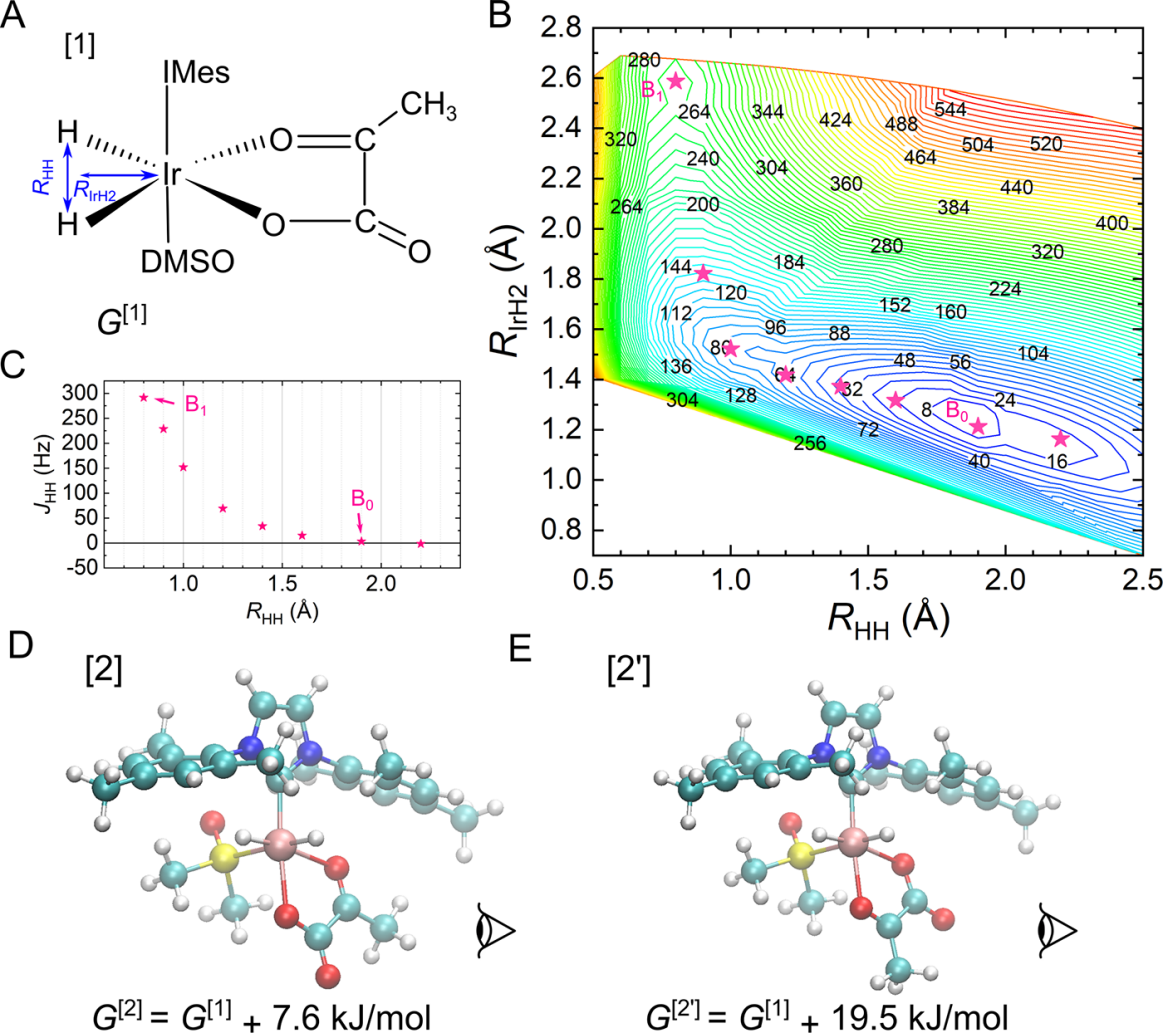
Quantum chemical calculations for three Ir complexes. (A) Chemical structure of complex [**1**] defining Ir–H_2_ and H–H distances. (B) Relaxed relative potential energy surface of complex [**1**] along the H–H and Ir–H_2_ distances, with contour lines increasing in 8 kJ/mol intervals. (C) Computed *J*_H–H_ values when moving from dihydrogen to the dihydride complex (denoted with stars) on the PES. (D and E) Three-dimensional chemical structures and energies for complexes [**2**] and [**2′**], respectively. The computed *J* couplings are as follows: *J*_HH_^[**1**]^ = −0.041 Hz, *J*_HH_^[**2**]^ = 2.29 Hz, and *J*_HH_^[**2′**]^ = 0.912 Hz. The measured values are as follows: *J*_HH_^[**1**]^ = −10.48 Hz, and *J*_HH_^[**2**]^ = −6.55 Hz. The eye icons highlight the region of pyruvate reorientation.

According to a previous study by Gelabert et al. on a similar iridium hydride complex,^38^ the Ir–H_2_ unit exhibits complex dynamics complicated by the motions of the two hydrogen atoms bound to Ir, giving rise to both dihydrogen and dihydride complexes in extreme cases. Therefore, a two-dimensional surface scan was performed for complex [**1**] (Figure 2B), where the H–H (*R*_HH_) and Ir–H_2_ distances (*R*_Ir–H_2__, the distance between Ir and the midpoint between the two hydrogen atoms) distances were varied while the Ir–H^a^ and Ir–H^b^ were kept equal (Figure 2A).

The energy minimum structure is a dihydride complex (*R*_HH_ = 1.9 Å). Another very shallow minimum shows a certain degree of dihydrogen character (*R*_HH_ = 1.2 Å), which is, however, 64 kJ/mol higher in energy. Both structures are true minima without imaginary frequencies. When we move from dihydrogen (*R*_HH_ = 0.8 Å) to the dihydride complex (*R*_HH_ = 1.9 Å), the corresponding *J*_HH_ value decreases rapidly as the H-H bond breaks (Figure 2C). Note that experimentally, a negative *J*_HH_ value is observed for the two now inequivalent hydride ligands; negative values were found for the dissociated hydrogens (*R*_HH_ > 1.9 Å). Overall, the shallow potential energy surface implies that a more accurate estimate of the *J*_HH_ value should be calculated using a Boltzmann average of all possible vibrational states at a given temperature in the anharmonic potential well and include nonclassical dynamics, the influence of a heavy transition metal atom, and its complex electronic structure.^38^ Hence, while structures and relative energies for such compounds are typically reasonable, the computed *J* coupling and chemical shift values are, as expected (Figure 2C and Tables S4 and S5), not accurate enough to replicate the experimental observations.

To measure these *J* couplings experimentally, one can analyze the ^1^H–^13^C *J* couplings by measuring ^1^H NMR spectra of solutions containing the activated IrIMes catalyst with sodium 1-^13^C-pyr or 2-^13^C-pyr and DMSO-*d*_6_ in methanol-*d*_4_ after the addition of pH_2_ to the solution. Upon hydrogen exchange and addition of pH_2_ to IrIMes, three pairs of antiphase resonances for the hydride ligands in Ir complexes [**1**]–[**3**] (Figure 1B) are observed. Such an NMR spectrum and the corresponding spin order are often termed under PASADENA (parahydrogen and synthesis allow dramatically enhanced nuclear alignment) conditions.^39^ Note that [**1**] and [**2**] are regioisomers for which in [**1**], pyr coordinates *trans* to hydride, and in [**2**], pyr is *trans* to hydride and NHC; complex [**3**] contains no pyr. The result of these arrangements is that the hydride ligands are chemically inequivalent, and we will refer to them as H^a^ and H^b^ as depicted in Figure 1A. Before, the chemical shifts of the pair of hydride ligands were assigned to the corresponding molecular composition (not specifying which of two hydrides has which chemical shift),^15^ while here, we tentatively assign the chemical shifts of protons to the nuclei in the complex as discussed below.

By bubbling pH_2_ through the methanol-*d*_4_ solution at a high magnetic field (9.4 T), we obtained ^1^H PASADENA type NMR spectra (Figure 1B), where visual inspection yields only the H^a^–H^b^ coupling constants: *J*_HH_^[**1**]^ = −10.5 Hz, *J*_HH_^[**2**]^ = −6.5 Hz, and *J*_HH_^[**3**]^ = −6.15 Hz. However, no visible interactions with ^13^C were seen, except for H^b^ to 2-^13^C-pyr in [**2**].

Applying ^13^C decoupling narrowed some lines in the ^1^H{^13^C} PASADENA spectra (Figure 1B), which let us estimate ^1^H–^13^C interactions. It proved to be possible to discern that C^1^ has the strongest interaction with H^a^ in [**1**], while C^2^ has the strongest interaction with H^b^ in [**2**] and negligible interaction with H^b^ [**1**]:  ≅ 0.9 Hz,  ≅ 0.8 Hz,  ≅ 1.6 Hz, ≅ 0.2 Hz,  ≅ 0.4 Hz,  ≅ 0.2 Hz,  ≅ 1.1 Hz, and  ≅ 2.7 Hz (see Figures S6–S8). However, this method is not very reliable because its precision relies on a reproducible *T*_2_* (compromised by gas bubbling). Therefore, we propose to use SOT sequences to estimate these small interactions.^40^

The efficiency of RF-based SOT sequences is strongly dependent on the interplay of *J* couplings and sequence timings. Thus, the *J* couplings can be determined by varying the timings and recording the polarization.

SOT in SABRE experiments, however, is challenging due to the interplay of chemical exchange and *J* coupling interactions.^41,42^ The complex needs to exist for the duration of the SOT sequence. Cooling a sample reduced the bond rupture rate, prolonging the complex’s lifetime and the viable period for SOT.^16,43^ The maximum ^1^H PASADENA signal intensity for complex [**1**] was achieved at 267 K [decreasing further for lower temperatures (Figure S1)]. As [**1**] is assumed to be responsible for producing hyperpolarized pyr, it is the most important of the three complexes. Hence, we probed the *J* coupling interactions of [**1**] at this temperature (and lower temperatures) to decrease the exchange rate and maximize polarization.^15^

We started with the pH_2_ and insensitive nuclei polarization transfer sequence (phINEPT),^44^ which has already been used for SABRE to enhance ^15^N signals (and will be used for ^13^C in this work) and to measure lifetimes for Ir complexes.^43,45^ This approach theoretically allows one to probe the *J* couplings. However, when the simple phINEPT approach is used, it becomes apparent that it is virtually impossible to simultaneously measure two weak ^1^H–^13^C *J* coupling constants (Figure S5).

Instead, we hypothesized that frequency-selective excitations^46^ can be used to probe individual *J* coupling interactions between one of the protons and one of the ^13^C nuclei. To disentangle all of these interactions, we employ frequency-selective excitation of polarization with PASADENA (SEPP) followed by insensitive nuclei enhanced by polarization transfer (INEPT) with selective pulses (SP); altogether, this is a frequency-selective version of SEPP-INEPT^47^ [SEPP-SPINEPT (Figure 3)].^48^ In this sequence, we set the excitations such that no more than two spins are excited simultaneously during SOT, enabling us to probe interactions between two spins only.

**Figure 3:**
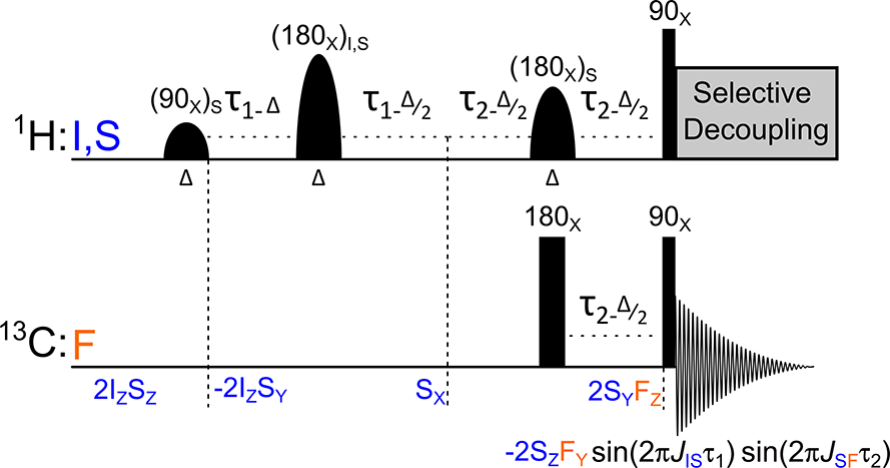
Scheme of ^1^H–^13^C selective spin order transfer sequence SEPP-SPINEPT. The spin evolution of two protons (I and S spins) and one ^13^C (F spin) is indicated. As a result, an antiphase ^13^C signal split by *J*_SF_ can be observed. The amplitude of such a signal is modulated with only two interactions as sin(2*πJ*_IS_τ_1_) sin(2*πJ*_SF_τ_2_). By fixing τ_1_ ≅1/(4*J*_IS_) and varying the duration of the τ_2_ interval, one can measure the corresponding ^1^H–^13^C interaction. The round pulses are frequency-selective pulses with different amplitudes and the same duration Δ. Additional indices for selective pulses indicate excited nuclear spins. Rectangle pulses indicate the hard pulses. During acquisition, a selective decoupling {^1^H} at 2.36 ppm was added to decouple the methyl group’s interaction. Due to rapid chemical exchange, the optimal τ_1_ can differ from 1/(4*J*_IS_) (examples in Figure S4).

A high-field SABRE experiment generates *I*_Z_^H^a^^*I*_Z_^H^b^^ spin order on the IrHH protons. When we probed for a *J*_H^a^C_ interaction, we exited H^a^ with the first selective pulse, generating the following spin order after SEPP-SPINEPT *I*_Z_^H^a^^*I*_Y_^C^ sin(2π*J*_HH_τ_1_) sin(2π*J*_H^a^C_τ_2_). Now, if τ_1_ is set to the optimal value [close to 1/(4*J*_HH_) (see Figure S4)] and τ_2_ is varied, the effect of *J*_H^a^C_ is mapped. This process was repeated for H^b^, the second ^13^C spin, and the other complexes to probe the remaining interactions. Optionally, one can accelerate this approach by the simultaneous excitation of complexes [**1**] and [**2**], so that two data points result per experiment. Multimode excitation was used recently for the generation of multiple long-lived spin states.^49^

By fitting the measured SEPP-SPINEPT kinetics with *A* sin(2π*J*_HC_τ_2_) exp(−2τ_2_*R*), one can estimate the corresponding ^1^H–^13^C interaction, where *R* = *k*_d_ + *R*_2_ is the superposition of the dissociation rate constant (1/*k*_d_ is the lifetime of the complex) and *R*_2_ is the effective transversal relaxation time constant. This description shows that SEPP-SPINEPT is useful for asymmetric systems as it needs chemically non-equivalent IrHH protons. However, it will not be practical for the IrIMes complex with three pyridine ligands, where IrHH are chemically equivalent.^14^

Using this approach, we transferred polarization to ^13^C^1^ and ^13^C^2^ spins in complexes [**1**] (Figure 4) and [**2**] (Figure 5) at three temperatures (256, 261, and 267 K) and estimated corresponding ^1^H–^13^C interactions (Table 1).

**Figure 4:**
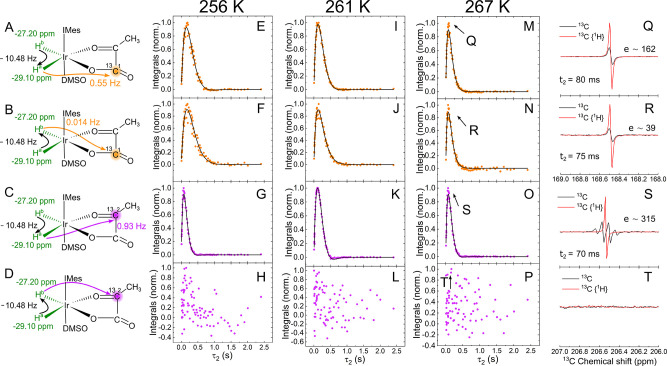
SEPP-SPINEPT applied to complex [**1**]. (A–D) Schemes represent selective SOT between IrHH protons (−29.10 and −27.20 ppm) and consequent polarization transfer to ^13^C^1^ (168.54 ppm) or ^13^C^2^ (206.59 ppm). (E–P) Normalized fitted integrals of absolute ^13^C signals of bound pyr after SEPP-SPINEPT as a function of τ_2_ and (Q–T) exemplary phased spectra at a maximum of polarization with signal enhancement (ε) at 267 K (black) and with {^1^H} decoupling (red). No polarization was observed when polarization was transferred from H^b^ to ^13^C^2^ (H and L). Phased spectra were integrated (dots) and fitted with *A* sin(2*πJ*_CH_τ_2_) exp(−2τ_2_*R*) (lines), giving decaying *R* and *J* coupling values (Table 1). The duration of selective pulses was 10 ms, and the first delay (τ_1_) was 20 ms.

**Figure 5:**
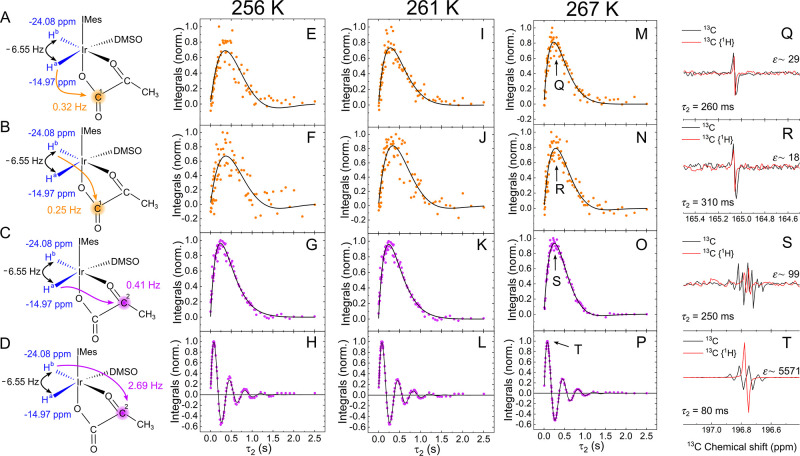
SEPP-SPINEPT applied to complex [**2**]. (A–D) Schemes represent selective SOT between IrHH protons (−24.08 and −14.97 ppm) and consequent polarization transfer to ^13^C^1^ (165.14 ppm) or ^13^C^2^ (196.86 ppm). (E–P) Normalized fitted integrals of ^13^C signals of bound pyr after SEPP-SPINEPT as a function of τ_2_ and (Q–T) exemplary phased spectra at a maximum of polarization with signal enhancement (ε) at 267 K (black) and with {^1^H} decoupling (red). Phased spectra were integrated (dots) and fitted with *A* sin(2*πJ*_CH_τ_2_) exp(−2τ_2_*R*) (lines), giving decaying R and J coupling values (Table 1). The duration of selective pulses was 10 ms, and the first delay (τ_1_) was 38 ms.

**Table 1:** ^1^H–^13^C Coupling Constants for Complexes [**1**] and [**2**] for Both Protons and C^1^ and C^2^ of pyr Measured with SEPP-SPINEPT (*) or SEPP-SPINEPT{^1^H} (^†^), Estimated before^27^ and ^1^H–^1^H Coupling Constants for Complexes [**1**]–[**3**] Measured with SEPP (‡, Figure S4)^a^

	[**1**]	[**2**]
δ_H^a^_ (ppm)	–29.10	–14.97
δ_H^b^_ (ppm)	–27.20	–24.08
δ_C^1^_ (ppm)	168.54	165.15
δ_C^2^_ (ppm)	206.59	196.86
*J*_H^a^–H^b^_ (Hz)	–10.48^‡^	–6.55^‡^
*J*_C^1^–H^a^_ (Hz)	|0.55 ± 0.02|*	|0.32 ± 0.03|*
	|0.53 ± 0.02|^†^	|0.21 ± 0.04|^†^
	–0.97^27^	
*J*_C^1^–H^b^_ (Hz)	|0.014 ± 0.00|*	|0.25 ± 0.03|*
	|0.006 ± 0.00|^†^	|0.025 ± 0.33|^†^
	0.8^27^	
*J*_C^2^–H^a^_ (Hz)	|0.93 ± 0.08|*	|0.41 ± 0.02|*
	|0.99 ± 0.016|^†^	|0.45 ± 0.02|^†^
	–0.5^27^	
*J*_C^2^–H^b^_ (Hz)	not observable*^†^	|2.69 ± 0.002|*
	–0.06^27^	|2.69 ± 0.035|^†^
*R*_*T*=256 K_ (s^–1^)	3.16 ± 0.03*	1.36 ± 0.05*
	2.96 ± 0.03^†^	1.47 ± 0.06^†^
*R*_*T*=261 K_ (s^–1^)	4.19 ± 0.04*	1.56 ± 0.05*
	4.06 ± 0.04^†^	1.58 ± 0.06^†^
*R*_*T*=267 K_ (s^–1^)	7.05 ± 0.07*	1.91 ± 0.05*
	6.73 ± 0.07^†^	1.96 ± 0.05^†^

a The *J*_HH_ values are −10.48 ± 0.02 Hz for complex [**1**], −6.55 ± 0.01 Hz for complex [**2**], and −6.03 ± 0.01 Hz for complex [**3**] (−21.59 and −15.56 ppm). Measurements were taken at three temperatures (256, 261, and 267 K). The reference for the ^13^C chemical shift was 47.63 ppm with methanol-*d*_4_.^36^ SEPP-SPINEPT does not provide any information about the sign of *J* couplings.

Exemplary SEPP-SPINEPT kinetics measured at 256, 261, and 267 K are shown in Figures 4 and 5. No signal was observed for ^13^C^2^ of [**1**] when polarization was transferred from H^b^, meaning that the *J* coupling is very small and is the smallest interaction among all for both complexes [**1**] and [**2**]. The H^b^–C^2^ interaction of [**2**] was visible in ^1^H PASADENA (Figure 1) and confirmed with SEPP-SPINEPT (Figure 5H), which equals ∼2.69 Hz and does not need additional verification.

The other six interactions (three for each complex) are <2 Hz but still facilitate transfer polarization. Cooling the sample to 256 K (the lowest available temperature for our system) allowed us to slow the chemical exchange and prolong the complex’s lifetime, enabling more accurate *J* coupling determinations by comparing measurements at different temperatures.

A global fitting approach was employed to enhance the accuracy of the fitting, allowing *J* coupling parameters and relaxation parameter *R* to be shared across the data sets (detailed in Table 1 and Figures S9 and S10). Variation of the temperature from 267 to 256 K more than halved effective relaxation parameter *R* for [**1**] from ∼7 to ∼3 s^–1^, while for complex [**2**], the reduction was <1 s^–1^, highlighting the contributions from the chemical exchange and differences between dynamics of the complexes.

In addition, parameter *R* in our fittings (Table 1) provides valuable estimates of exchange rates, which exhibit a strong correlation with temperature; however, it is important to acknowledge that these estimates can also be affected by the pressure-dependent association mechanism of H_2_ exchange and temperature-dependent *T*_1_ relaxation. Further investigations of the [H_2_] and [pyr] effects will be conducted separately.

Assuming that the effective relaxation of the system during the polarization transfer is ∼1 s^–1^ (typical order of magnitude for the relaxation time of hydride protons), the lifetimes at, e.g., 267 K are 0.16 s for complex [**1**] and 1 s for complex [**2**]. Using such relaxation estimates, the values for the enthalpy and entropy of activation are 43.4 kJ/mol and −64.39 J mol^–1^ K^–1^ for complex [**1**] and 41.7 kJ/mol and −88.47 J mol^–1^ K^–1^ for complex [**2**], respectively (Figure S11B).

In further studies, for an even better assessment of interactions and exchange, one should go to even lower temperatures, ∼250 K, where the lifetime of complex [**1**] is estimated to be similar to the spin–spin relaxation of the hydride protons or lower (see Figure S11A). Similar *R* values were obtained using the SEPP experiment (Table S1 and Figure S4).

The use of decoupling during the NMR analysis has emerged as a superior strategy, significantly outperforming the nondecoupled spectra. A selective ^1^H decoupling at 2.36 ppm allowed decoupling of the hydrogen interactions of the ethyl group. One can see a simplification of the spectrum at 206.59 and 196.86 ppm for 2-^13^C-pyr (Figures 3S,T and 4S,T). In addition, selective decoupling enhanced the accuracy of signal fitting by maximizing the intensity of the SEPP-SPINEPT signal.

Previously, all of these interactions were estimated to explain polarization transfer to 1-^13^C-pyr. For example, a 5 Hz interaction was estimated by Nantogma et al.,^17^ while the strongest interaction we found for [**1**] was only 0.55 Hz. In the other study,^31^ the minimal interaction of 0.06 Hz was assumed because no broadening of the ^13^C spectral lines was visible. However, as the authors explain,^31^ this effect may be caused by line narrowing due to rapid chemical exchange; our estimations of the chemical-exchange rates agree with this explanation. Complexes [**1**]–[**3**] are the primary complexes of the system; however, there are others visible in ^1^H PASADENA and ^13^C SEPP-SPINEPT (examples in Figure S12), indicating even greater complexity of this dynamic SABRE system.

The best estimates for the interactions so far were achieved by combining results of SOT at ultralow magnetic fields and DFT calculations.^27^ Our experimental methods allowed us to determine the magnitude but not the sign of the *J* coupling interactions. DFT calculations and low-field experiments shed some light on the signs of these interactions.^27^ We assigned the IrHH chemical shifts to the structure of [**1**] such that H^a^ (−29.10 ppm) and C^2^ (206.59 ppm) with the largest *J* coupling of 0.93 Hz are in the *trans* position; however, this is a tentative assignment, and the measurements of distances using nuclear Overhauser effects would give better structure evaluation.

The selective nature of SEPP-SPINEPT excitation sequences addresses the issue of dynamic exchange as it creates two-spin order coherence, which, in addition to decaying through relaxation, will vanish as the Ir–O^13^C bond to pyr breaks (the IrHH bonds in these complexes rupture after this step). Consequently, the resulting signal oscillation will experience a modulation that will dampen its amplitude, limiting the *J* coupling size that can be measured at any particular temperature. This reduction at the temperatures (and, therefore, bond rupture rate) selected has a minimal effect on the position of the peak maximum, which defines the measured coupling as the rate of signal damping acts on a longer time scale. Furthermore, deploying hyperpolarization helps address this detail by increasing the signal-to-noise ratio. Therefore, this approach is robust, overcoming the inherent challenges when dealing with the dynamic behavior of these critical Ir complexes. The obtained NMR parameters of the short-lived Ir complexes can be used as a benchmark for quantum chemical calculations of hydrogens near heavy metals. Also, the parameters obtained here can be used to numerically optimize SOT for even stronger hyperpolarization of pyr with SABRE. This method and these results will greatly facilitate further development of the up-and-coming SABRE technology.

## Methods

*Chemicals*. Perdeuterated Ir precatalyst [Ir-*d*_22_] = [IrCl(COD)(IMes-*d*_22_)] was synthesized according to ref (50) [IMes = 1,3-bis(2,4,6-trimethylphenyl)imidazol-2-ylidene, and COD = cyclooctadiene], and sodium pyruvate-1-^13^C (^13^C-pyr, 490709, Sigma-Aldrich), sodium pyruvate-2-^13^C (^13^C-pyr, 490725, Sigma-Aldrich), dimethyl sulfoxide-*d*_6_ (DMSO, 00905-25, DEUTERO GmbH), and methanol-*d*_4_ (441384, Sigma-Aldrich) were used here.

*Sample*. Samples were prepared with a fixed ratio of substrate, catalyst, and DMSO in 450 μL of methanol-*d*_4_ and placed in a high-pressure 5 mm NMR tube (524-PV-7, DEUTERO GmbH). The ratio of the substrate, catalyst (5 mM), and DMSO was 10:1:5.

*Experiment*. The NMR tube was then attached to a bubbling system (similar to that used in ref (51)) and placed inside the spectrometer (Bruker Neo, 9.4 T). The solution was bubbled with parahydrogen-enriched H_2_ gas to a pressure of 8.7 bar. The gas was enriched to >92% pH_2_ in the presence of a spin-exchange catalyst (Fe_2_O_3_) at a low temperature (25 K). During acquisition, a selective decoupling {^1^H} of 250 Hz at 2.36 ppm was applied for 1 ms by using WALTZ-16 with a *P* of 0.0024 W.

*Fitting*. ^1^H PASADENA and SEPP-SPINEPT kinetics were fitted using MATLAB scripts (available in the Supporting Information). The details of the fitting functions and global fittings are available in the Supporting Information. All error margins for the fitted values are standard deviations estimated using the MATLAB nonlinear regression “nlinfit” function.

*Computational Details*. All quantum chemical calculations were performed with the ORCA 5.0 program package.^52^ Geometries were optimized at the B3LYP-D4/def2-TZVP level of theory.^53−56^ The optimized structures were confirmed to be true minima by harmonic vibrational frequency analyses. The NMR chemical shifts were calculated at the GIAO-ZORA-TPSSh/def2-TZVPP level of theory,^57−61^ and the coupling constants were calculated at the PBE/pcJ-3 level of theory,^62,63^ where the SARC-ZORA-TZVPP basis set was used for Ir^64^ and the def2-TZVPP or pcJ-3 basis set was used for all other nuclei. All basis sets were decontracted and complemented with the “Autoaux” auxiliary basis sets available in ORCA.^65^ Very tight convergence criteria were employed for the optimization and self-consistent field, and a tight “DefGrid3” DFT integration grid was applied. The CPCM model was used in both geometry optimizations and NMR calculations to account for solvation effects.^66^

## Data Availability

The corresponding raw data can be accessed via Zenodo https://zenodo.org/doi/10.5281/zenodo.10512816.

## Supporting Information Available

Additional ^1^H and ^13^C NMR spectra, SEPP and phINEPT polarization transfer sequences, and estimation of *J* coupling constants using ^1^H and ^1^H{^13^C} PASADENA spectra (PDF)
SEPP-SPINEPT fitting matlab scripts (ZIP)
Geometries of identified complexes (ZIP)
Transparent Peer Review report available (PDF)
