# Supplementary material for: J Coupling Constants of <1 Hz Enable 13C Hyperpolarization of Pyruvate via Reversible Exchange of Parahydrogen
Source: J Phys Chem Lett. 2024 Jan 25;15(5):1195–203. doi: 10.1021/acs.jpclett.3c02980 (PMC10860132; doi:10.1021/acs.jpclett.3c02980)
Supplement: Supplementary file 1 — jz3c02980_si_001.pdf [file jz3c02980_si_001.pdf]

J Coupling Constants of <1 Hz Enable ^{13}C Hyperpolarization Of Pyruvate Via Reversible Exchange Of Parahydrogen

Charbel D. Assaf[*1], Xin Gui[2], Alexander A. Auer[2], Simon B. Duckett[3], Jan-Bernd Hövener[1], Andrey N. Pravdivtsev*[*1]*

[1] Section Biomedical Imaging, Molecular Imaging North Competence Center (MOIN CC), Department of Radiology and Neuroradiology, University Medical Center Kiel, Kiel University, Am Botanischen Garten 14, 24118, Kiel, Germany, E-mail: jan.hoevener@rad.uni-kiel.de

[2] Max-Planck-Institut für Kohlenforschung, Kaiser-Wilhelm-Platz 1, 45470 Mülheim an der Ruhr, alexander.auer@kofo.mpg.de

[3] Centre for Hyperpolarization in Magnetic Resonance (CHyM), University of York, Heslington YO10 5NY, UK, E-mail: simon.duckett@york.ac.uk

Corresponding Authors

* charbel.assaf@rad.uni-kiel.de, andrey.pravdivtsev@rad.uni-kiel.de,.

Contents

^{13}C enhancement and polarization S-3
Temperature dependence of ^{1}H PASADENA signal for three complexes S-4
Optimization of the power of the selective pulses S-5
SEPP optimization S-6
Effect of selective pulse length on SEPP S-7
phINEPT pulse sequence S-8
Estimation of J_{CH} from ^{1}H PASADENA spectra S-9
Estimation of J_{CH} for complex [1] and [2] for both 1,2-^{13}C-pyruvate using SEPP-SPINEPT. S-12
Estimation of J_{CH} for complex [1] and [2] for both 1,2-^{13}C-pyruvate using SEPP-SPINEPT with {^{1}H} decoupling S-14
Estimated temperature of dissociation rate S-15
Other hyperpolarized complexes S-16
Simulation of exchange with noise S-18
Quantum chemical calculations of the complex structures [1-3] and NMR parameters S-19
References S-21

^{13}C enhancement and polarization

The enhancement factor of the bound substrate was calculated using the signal intensities of the thermal and hyperpolarized pyruvate spectra:

$$\varepsilon = \frac{^{13\mathrm{C}}I^{\mathrm{HP}}}{^{13\mathrm{C}}I^{\mathrm{TP}}} \times \frac{RG^{\mathrm{TP}}}{RG^{\mathrm{HP}}} \times \frac{NS^{\mathrm{TP}}}{NS^{\mathrm{HP}}} \qquad \textbf{\textit{(Eq. S1)}}$$

where $^{13\mathrm{C}}I^{\mathrm{HP}}$ is the integral of the hyperpolarized ^{13}C signal, $^{13\mathrm{C}}I^{\mathrm{TP}}$ is the integral of the same of the same but thermally polarized ^{13}C signal, RG^{HP} and RG^{TP}, NS^{HP} and NS^{TP} are corresponding receiver gains and the number of scans used to acquire hyperpolarized and thermally polarized signals.

Corresponding polarization was calculated as follows:

$$P = \varepsilon \frac{\gamma_{13C} B_0 \hbar}{2\, k_B T} \qquad \textbf{\textit{(Eq. S2)}}$$

where γ_{13C} is the gyromagnetic ratio of ^{13}C, B_0 is the observation magnetic field, T is the temperature, $\hbar$ is the reduced Planck's constant, and k_B is Boltzmann's constant.

Temperature dependence of ^{1}H PASADENA signal for three complexes

The SOT in SABRE experiments is challenging due to the interplay of chemical exchange and spin-spin interactions. Therefore, temperature plays an important role in SABRE. After acquiring the ^{1}H PASADENA spectrum, one can notice three primary iridium complexes (**Figure 1**). Measuring the signal intensities of ^{1}H PASADENA, we found that the highest polarization for complex [**1**] and [**2**] is reached at 267 K (**Figure S1**). The sample composition was [Ir] = 5 mM, DMSO = 20 mM, [Pyr].= 50 mM in 400 µl of methanol-*d*4.

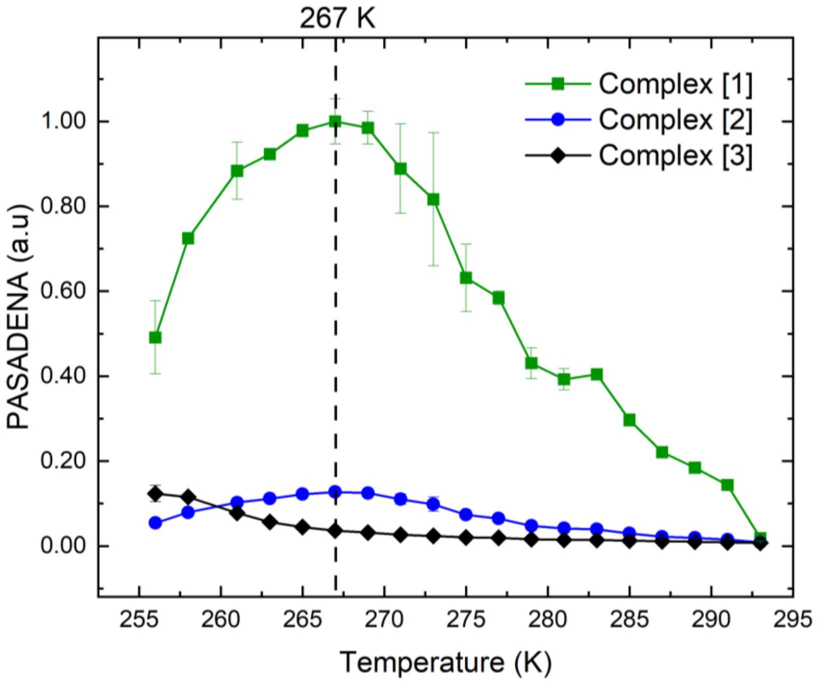

Figure S1. ^{1}H PASADENA of three primary complexes (Figure 1) as a function of temperature. At 267 K, the PASADENA signal of complex [**1**] reaches its maximum.

Optimization of the power of the selective pulses

We used selective pulses (SP) with the "Gaus1_180r.1000" shape available in TopSpin with 8, 10, and 12 ms duration. In this section, we describe how we calibrated the power of 10 ms SP pulses. Using the pulse sequence consisting of SP – broad band 90° pulse – acquisition of free induction decay (FID), we measured the signal as a function of the amplitude of SP (**Figure S2**).

For an SP with one carrier frequency (SP-1) and a length of 10 ms, 90° is reached at 0.08147 V (0.00013285 W, 38.77 dB) and 180° at a double amplitude of 0.16311 V (0.0005315 W, 32.74 dB).

To excite two hydrogens of [**1**] (-27.2 ppm and -29.1 ppm) or [**2**] (-14.97 ppm and -24.08 ppm), we combined two SP with the same shape and different carrier frequencies, e.g., for complex [**1**] the first at 0 Hz and the second at 761.88 Hz (1.9 ppm to the left or to the right). The resulting SP with two carrier frequencies is called SP-2.

To achieve 180° with SP-2, two times higher amplitude than in SP-1 for 180° 0.3315 V (0.0021979 W, 26.58 dB).

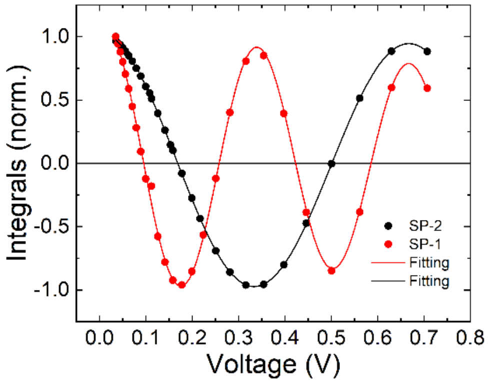

Figure S2. NMR signal after application of selective pulse (SP) with one carrier frequency (SP-1, red) and two carrier frequencies (SP-2, black). The sequence consisted of frequency selective pulse - broadband 90° pulse - acquisition of free induction decay (FID). The duration of SPs was 10 ms. SP-1 pulse with Gaus1_180r.1000 shape reached the 90° excitation at 0.08147 V (0.00013285 W, 38.77 dB) and 180° excitation at 0.16311 V (0.0005315 W, 32.74 dB). The SP-2 pulsed reached 180° excitation at 0.3315 V (0.0021979 W, 26.58 dB).

SEPP optimization

SEPP SOT converts the PASADENA two-spin order into the magnetization of one of them. We set out to find the delays τ and τ_3 (**Figure S3A**) of the pulse sequence that provides the highest signal.

We measured the SEPP signal for τ_3 of 0, 2.5, 5, and 7.5 ms, the duration of the SPs was Δ= 10 ms, and τ was changing from 0 to 100 ms. In total, 140 experiments were carried out (**Figure S3B**). The maximum signal was achieved at τ = 10 ms and τ_3 = Δ/2 = 5 ms. One can explain that adding τ_3 allows the proper rephasing of the spins. Below, we will show that SEPP performance is identical when using different durations of RF pulses (**Figure S4**).

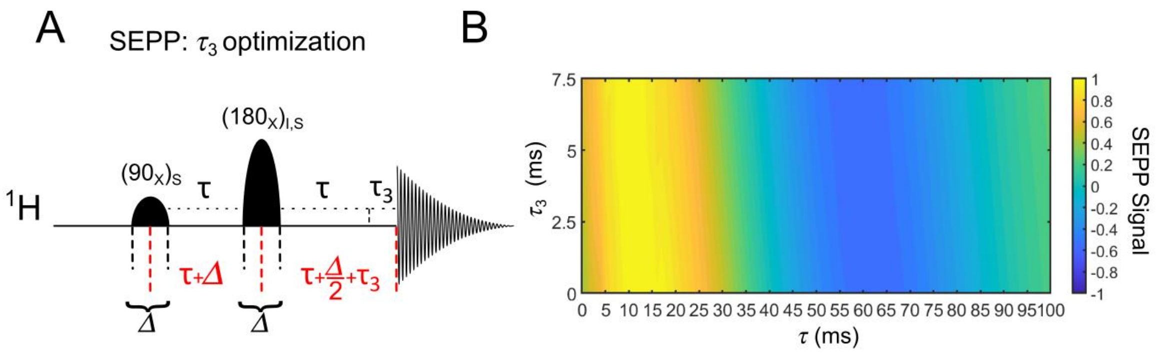

Figure S3. Optimization of SEPP SOT. SEPP sequence (A), the magnetization of H^a (-29.1ppm) of **[1]** after SEPP as a function of τ_3 and τ (B). The highest polarization achieved was at τ = 10 ms, τ_3 = 5 ms, and Δ = 10 ms. τ was varied in a step of 1 ms between 0 and 20 ms and in a step of 2 ms between 20 and 100 ms. The highest signal was achieved at τ = 10 ms and τ_3 = $\Delta/2$ = 5 ms.

Effect of selective pulse length on SEPP

Using SEPP, one can transfer the PASADENA spin order into the magnetization of one of the spins. Here, we analyzed the effect of the pulse length on SEPP performance (**Figure S4**). We repeated SEPP with three different pulse lengths of 8 ms, 10 ms, and 12 ms in function of τ_1 (**Figure S4A**); no significant difference was found. The maximum for complex [1] at 267 K was reached at $\tau_1 \cong 20$ ms $\cong \frac{1}{4J_{\mathrm{HH}}^{[1]}} =$ 23 ms, for complex [2] at $\tau_1 \cong 38$ ms $\cong \frac{1}{4J_{\mathrm{HH}}^{[2]}} = 38$ ms and for complex [3] at $\tau_1 \cong 29$ ms $\cong \frac{1}{4J_{\mathrm{HH}}^{[3]}} =$ 41 ms. As one can see, due to signal decay, optimal τ_1 differs from predicted $\frac{1}{4J_{\mathrm{HH}}^{[2]}}$. As one can see, **[3]** has the fastest R and hence the largest deviation of experimentally optimal τ_1 from theoretically estimated. On contrast, the **[2]** has the slowest R and experimentally optimal τ_1 coincides with the theoretically predicted.

The integrals of the spectral lines were well fitted with a damped sine wave function: $A \times \sin(2\pi J_{\mathrm{HH}}\tau_1) \times \exp(-2\tau_1 \times R)$. The results of fit for three complexes are given in **Table S1**. The signal decay rate R is a superposition of relaxation and dissociation rate.

Table S1. Parameters of three complexes were measured with SEPP (Figure S4) and obtained by fitting the sine wave function. The sign of the interaction was found from ^{1}H PASADENA spectra.

	J_{HH} (Hz)	R (s^{-1})
**[1]**	-10.48 ± 0.02	5.92 ± 0.08
**[2]**	-6.55 ± 0.01	2.07 ± 0.03
**[3]**	-6.03 ± 0.01	11.20 ± 0.07

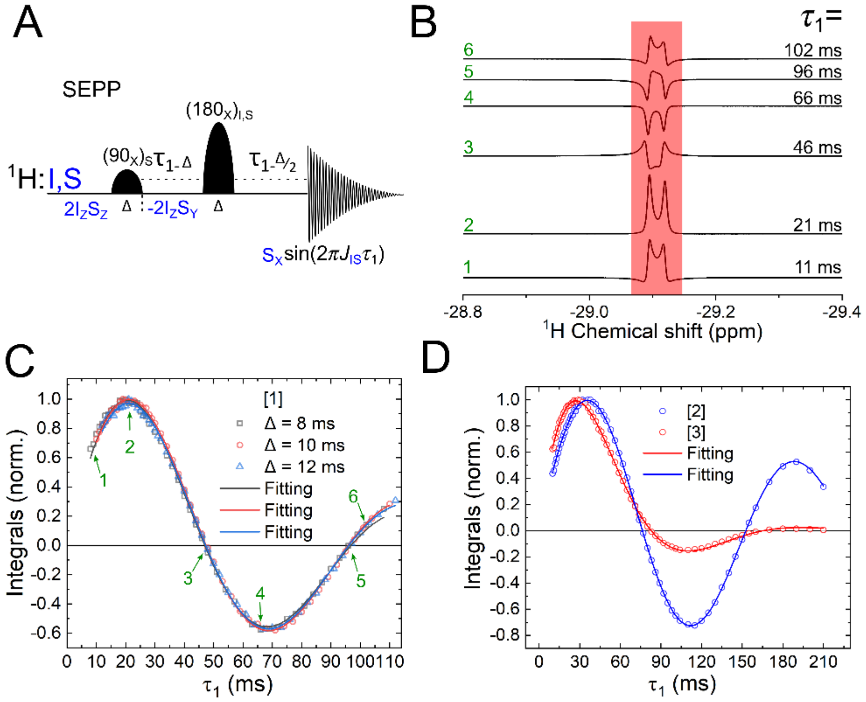

Figure S4. The effect of interpulse interval and pulse duration on the SEPP SOT. (A) Scheme of SEPP SOT. (B) ^{1}H SEPP spectra at some selected τ_1 values were obtained with 10 ms selective pulses. The red area indicates the integration region. (C) Integrals of polarized by SEPP proton of [**1**] as a function τ_1 for three durations of SPs: 8 (black), 10 (red), and 12 ms (blue). No significant difference was found. (D) Integrals of polarized proton of [**2**] (blue) and [**3**] (red). All experiments were carried out at 267 K at 9.4 T. The results of fit (C and D) are given in **Table S1**.

phINEPT pulse sequence

Application of phINEPT sequence was found to be impractical because the ^{13}C signal as a function of τ_1 is modulated by three interactions J_{HH}, $J_{\mathrm{H^aC}}$ and $J_{\mathrm{H^bC}}$ (**Figure S5**). Therefore, in the main text, we used frequency-selective SOT.

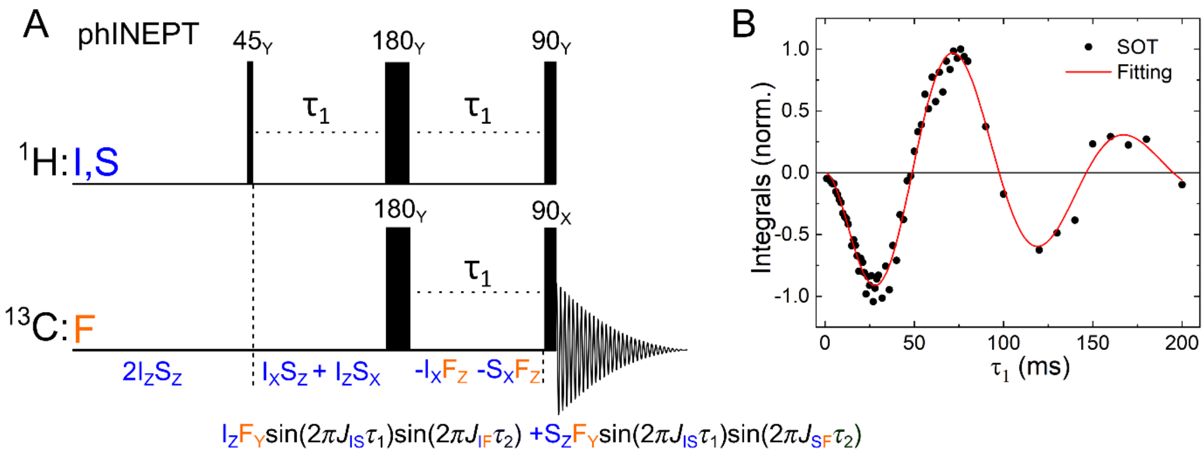

Figure S5. The phINEPT spin order transfer. (A) phINEPT sequences. Here only broadband RF pulses are used. (B) The integral of ^{13}C^1 signal in complex [1] as a function of τ_1. The weak ^{1}H-^{13}C interactions transfer the polarization from IrHH protons to primary evolution due to ^{13}C^1 in [1] but the signal is modulated by strong J_{HH} interactions that complicate the analysis. The spin orders after the application of phINEPT are depicted on (A).

Estimation of J_{CH} from ^1H PASADENA spectra

We compared the ^1H PASADENA spectra for [**1**] and [**2**] with either 1-^{13}C-pyr or 2-^{13}C-pyr (**Figures S6-8**). We noticed that full width at half maximum (FWHM) is larger in the spectrum without ^{13}C decoupling than in the spectrum with ^{13}C decoupling. This lets us estimate the ^{1}H-^{13}C interactions.

First, we measured ^{1}H PASADENA with ^{13}C decoupling (**Figure S6-8**, right-hand side). Then, we fit each spectrum using the sum of two Lorentzian functions:

$$\text{Spectrum} = \frac{A_1}{4(\delta-\delta_1)^2+FWHM_1^2} + \frac{A_2}{4(\delta-\delta_2)^2+FWHM_2^2} \quad \textbf{\textit{(Eq. S3)}}$$

The fit gave us two values of FWHM of both lines. Unfortunately, the two line widths were different due to the radiation-damping effect.

In the second step, we fit the ^{1}H PASADENA (**Figure S6-8**, left-hand side) with four Lorentzian functions using the same FWHMs that we evaluated before:

$$Spectrum = A_1 \times \left(\frac{1}{4\left(\delta-\delta_1+\frac{J_{CH}}{2}\right)^2+FWHM_1^2} + \frac{1}{4\left(\delta-\delta_1-\frac{J_{CH}}{2}\right)^2+FWHM_1^2}\right) + $$

$$+A_2 \times \left(\frac{1}{4\left(\delta-\delta_2+\frac{J_{CH}}{2}\right)^2+FWHM_2^2} + \frac{1}{4\left(\delta-\delta_2-\frac{J_{CH}}{2}\right)^2+FWHM_2^2}\right) \quad \textbf{\textit{(Eq S4)}}$$

Experimental data for 1-^{13}C-Pyr and 2-^{13}C-Pyr are plotted in **Figures S7-8** and the corresponding estimated J_{CH} are given in **Table S2**.

The fitting was implemented on MATLAB using nonlinear regression function "nlinfit". The fitting script, together with integrals, are available in Supporting Materials. The reported errors are the results of such fitting.

Table S2. Estimated J_{CH} interactions using broadening of ^{1}H spectrum due to small ^{13}C interactions.

	H^a	H^b
1-^{13}C-pyr [1]	0.896 ± 0.02	0.768 ± 0.02
2-^{13}C-pyr [1]	1.600 ± 0.09	0.245 ± 0.03
$\delta_{1\mathrm{H}}$ (ppm)	-29.1	-27.2
1-^{13}C-pyr [2]	0.408 ± 0.03	0.176 ± 0.07
2-^{13}C-pyr [2]	1.112 ± 0.04	2.698 ± 0.002
$\delta_{1\mathrm{H}}$ (ppm)	-14.97	-24.08

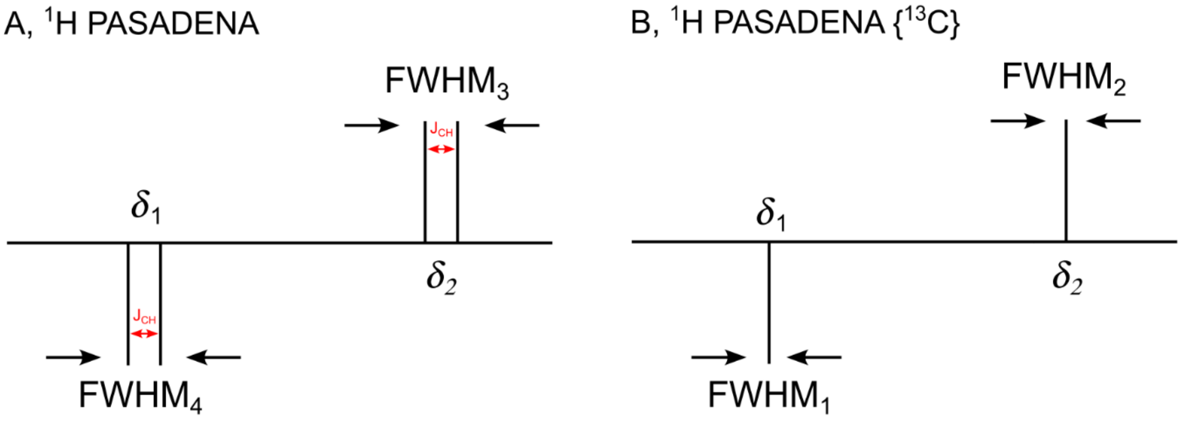

Figure S6. Stick spectra of ^{1}H PASADENA spectra without (A) and with ^{13}C decoupling (B). We measured the natural linewidths with ^{1}H PASADENA{^{13}C} spectrum and then used those values and **eq. S3-4,** we estimated J_{CH} interactions. As a result of these small interactions the linewidths in ^{1}H PASADENA are normally broader than in ^{1}H PASADENA {^{13}C}. The corresponding NMR spectra for complex [**1**] and [**2**] are given in **Figures S7** and **S8**. The estimated interactions are given in **Table S2**.

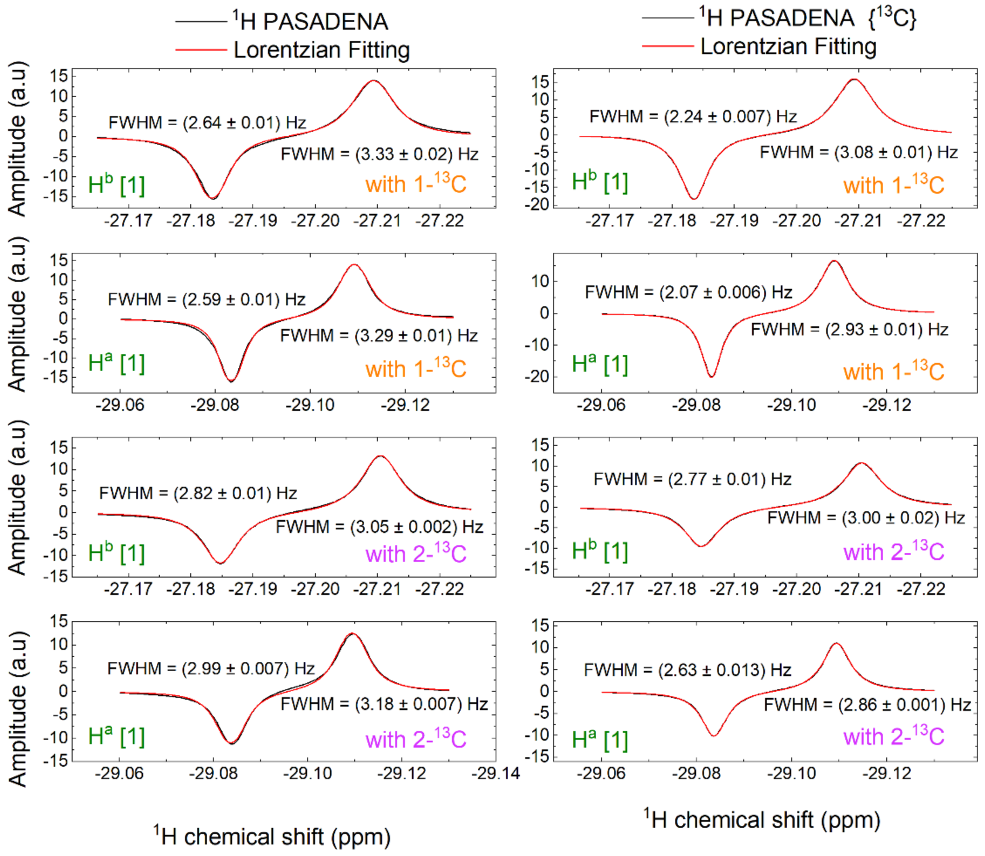

Figure S7. ^{1}H PASADENA (left column) and ^{1}H PASADENA {^{13}C} (right column) spectra for complex [1] with 1-^{13}C-Pyr (first two rows) and with 2-^{13}C-Pyr (bottom two rows).

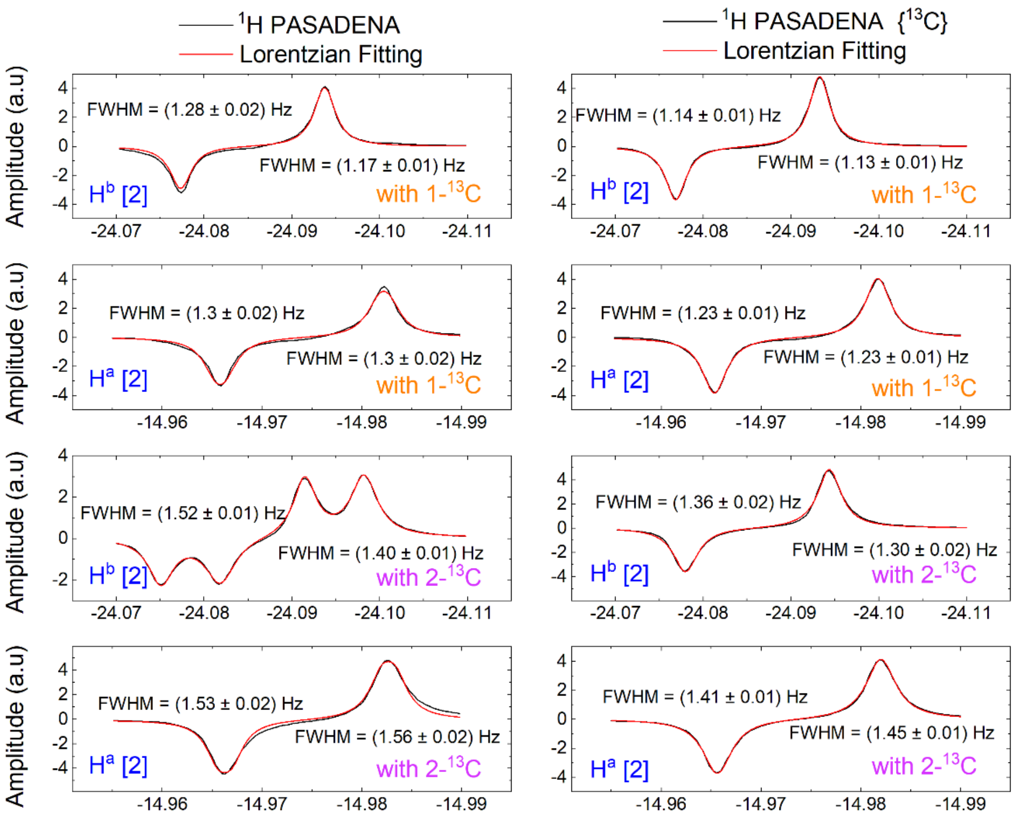

Figure S8. ^{1}H PASADENA (left column) and ^{1}H PASADENA {^{13}C} (right column) spectra for complex [2] with 1-^{13}C-Pyr (first two rows) and with 2-^{13}C-Pyr (bottom two rows).

Estimation of J_{CH} for complex [1] and [2] for both 1,2-^{13}C-pyruvate using SEPP-SPINEPT.

We employed the SEPP-SPINEPT pulse sequence for each hydride in complexes [1] and [2], and both 1 and 2-^{13}C pyruvate, and conducted experiments at three temperatures: 256, 261, and 267 K. Notably, H^b (-27.2 ppm) in 2-^{13}C complex **[1]** remained unobservable, while H^b (-24.09 ppm) in 2-^{13}C complex **[2]** exhibited a coupling of 2.69 Hz, effectively measured using ^{1}H PASADENA. For all observable ^{1}H-^{13}C interactions, we performed kinetic analysis, fitting the data with the equation A × sin($2\pi J_{CH}\tau_2$)×exp($-2\tau_2 R$), where J_{CH} represents the *J* coupling between proton and carbon, and *R* denotes the combined relaxation and exchange parameter. Each hydride possesses its distinct J_{CH} and exchange constant for each temperature. Notably, hydrides within the same complex share the same constant *R*, similar to the one measured with the SEPP (**Table S1**). To streamline the analysis, we employed a global fitting approach using MATLAB scripts to fit all parameters simultaneously using 9 or 12 kinetics for each complex (depending on the observable kinetics). Below, we show the fit when we used only 9 kinetics for each complex. Across all nine kinetics, we share nine amplitudes denoted as A_1 to A_9, three relaxation-exchange parameters *R* for each temperature, and three unique *J* coupling interactions.

To help understand the applied global fitting and parameters, see Table S3. Table S9 illustrates which parameters were used and were the same for some kinetics.

The reported errors are the results of such fits using MATLAB nonlinear regression function "nlinfit". The fitting script, together with integrals, are available in Supporting Materials.

Table S3. The sets of parameters used to fit SEPP-SPINEPT kinetics. See that *J*s are the same for each C-H pair, while *R*s are the same for each T.

SEPP-SPINEPT	T = 256 K	T = 261 K	T = 267 K
1-^{13}C-pyruvate, [1]-H^a	Set 1 (A_1, $J^{[1]}_{H^aC^1}$, $R^{[1]}_{256}$	Set 2 (A_2, $J^{[1]}_{H^aC^1}$, $R^{[1]}_{261}$)	Set 3 (A_3, $J^{[1]}_{H^aC^1}$, $R^{[1]}_{267}$)
1-^{13}C-pyruvate, [1]-H^b	Set 4 (A_4, $J^{[1]}_{H^bC^1}$, $R^{[1]}_{256}$)	Set 5 (A_5, $J^{[1]}_{H^bC^1}$, $R^{[1]}_{261}$)	Set 6 (A_6, $J^{[1]}_{H^bC^1}$, $R^{[1]}_{267}$)
2-^{13}C-pyruvate, [1]-H^a	Set 7 (A_7, $J^{[1]}_{H^aC^2}$, $R^{[1]}_{256}$)	Set 8 (A_8, $J^{[1]}_{H^aC^2}$, $R^{[1]}_{261}$)	Set 9 (A_9, $J^{[1]}_{H^aC^2}$, $R^{[1]}_{267}$)

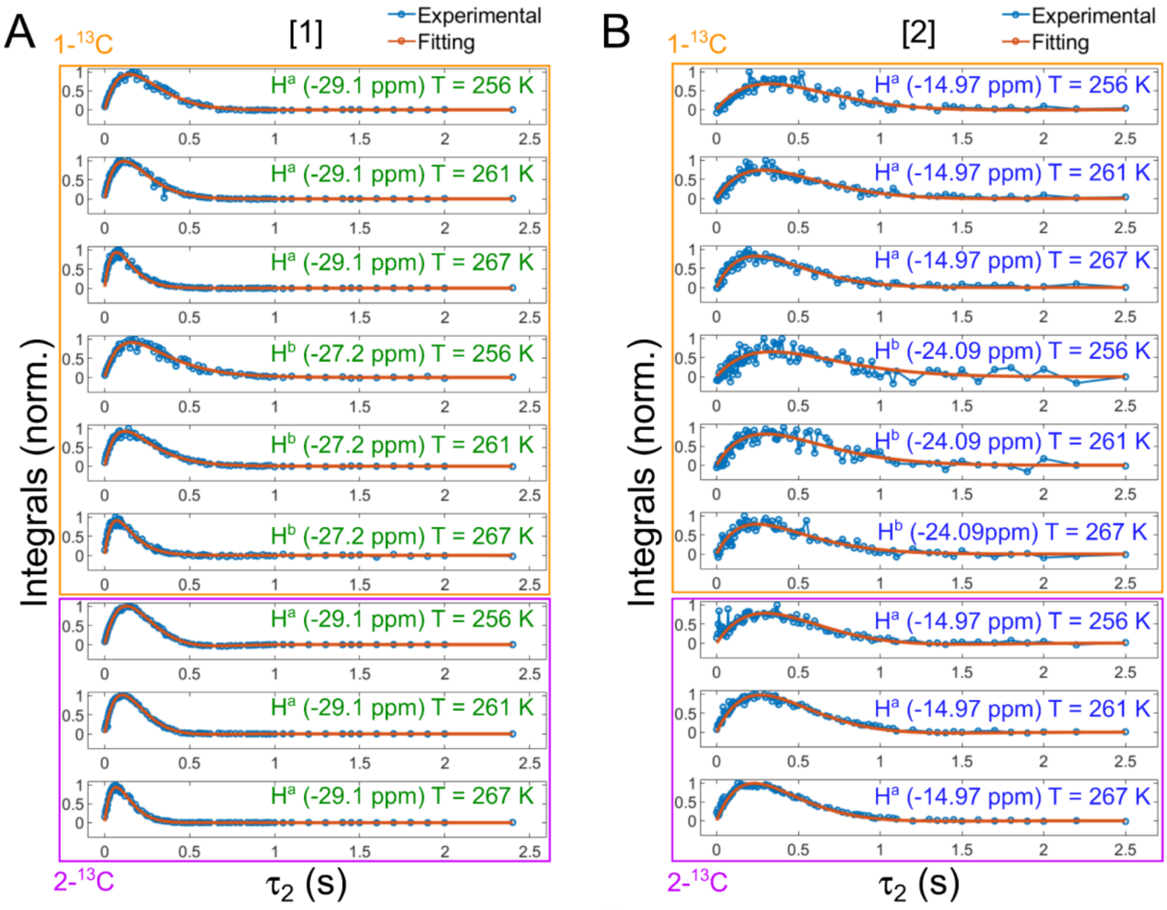

Figure S9. Fitting of SEPP-SPINEPT kinetics for complex [1] and [2] for both 1,2-^{13}C-pyruvate. (A) SEPP-SPINEPT kinetics for complex [1] and complex [2] (B), the first 6 rows are for 1-^{13}C-pyruvate, and the last 3 rows are for 2-^{13}C-pyruvate. All kinetics of each complex were fitted simultaneously, having common relaxation-exchange parameter *R* and J-coupling interactions.

Estimation of J_{CH} for complex [1] and [2] for both 1,2-^{13}C-pyruvate using SEPP-SPINEPT with {^{1}H} decoupling

We employed the SEPP-SPINEPT pulse sequence with {^{1}H} decoupling for each hydride in complexes [1] and [2], and both 1 and 2-^{13}C pyruvate, and conducted experiments at three temperatures: 256, 261, and 267 K. Same fitting process as in **Figure S9** was applied again here.

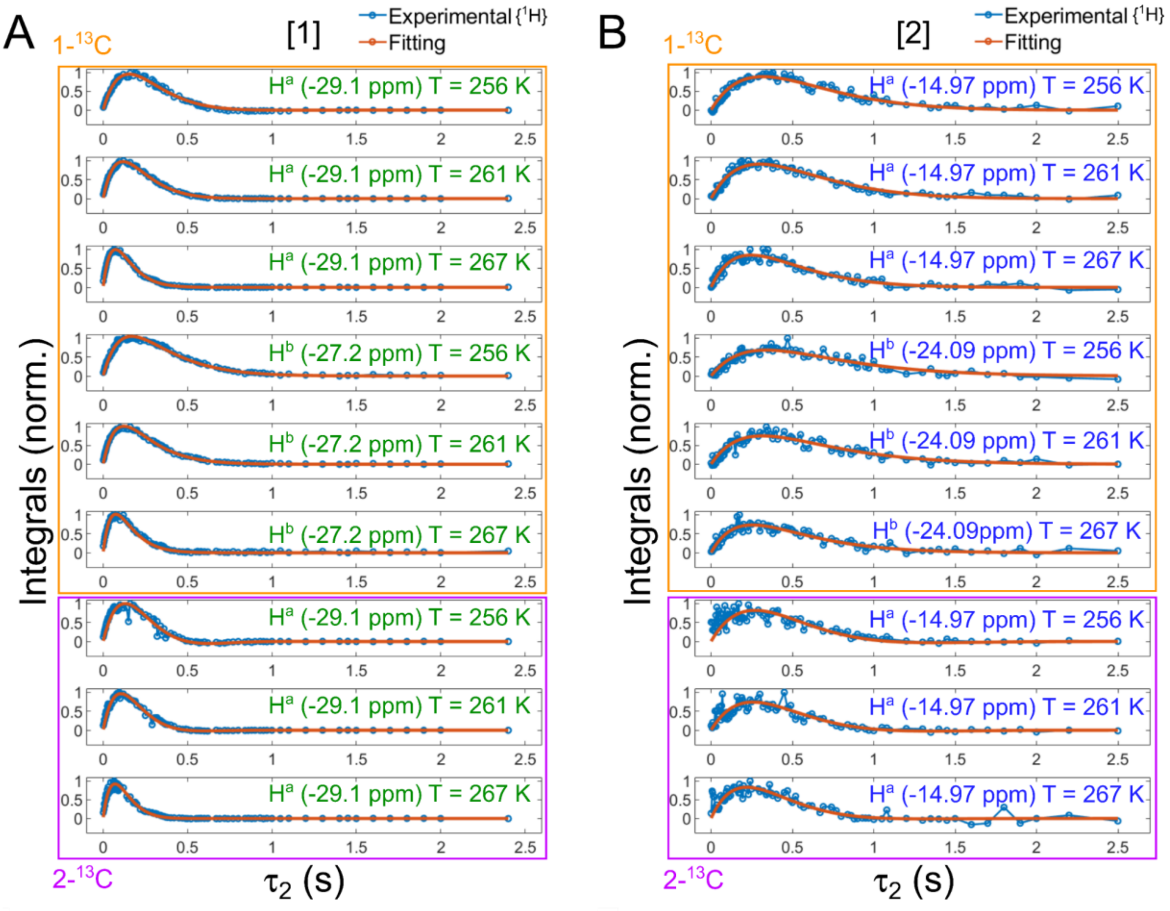

Figure S10. Fitting of SEPP-SPINEPT kinetics with {^{1}H} for complex [1] and [2] for both 1,2-^{13}C-pyruvate. (A) SEPP-SPINEPT kinetics {^{1}H} for complex [1] and complex [2] (B), the first 6 rows are for 1-^{13}C-pyruvate, and the last 3 rows are for 2-^{13}C-pyruvate. All kinetics of each complex were fitted simultaneously, having common relaxation-exchange parameter R and J coupling constants.

Estimated temperature of dissociation rate

Measuring J coupling less than 1 Hz is challenging in the presence of fast exchange. One can decrease the temperature to reduce the exchange rate to slow down the exchange rate. Using our SEPP-SPINEPT kinetics at temperatures 256, 261, and 267 K, we estimated the parameter $R(T) = k_d(T) - R_2(T)$. The typical relaxation rate of IrHH protons is about 1 s, hence if we estimate R_2 = 1 s^{-1} then we can approximate the exchange rates to lower temperatures using Arrhenius's equation:

$$k_d = Ae^{-\frac{E_a}{RT}} \quad \textbf{(Eq. S5)}$$

Which is convenient to fit with a linear function as follows:

$$\ln(k_d) = \ln(A) - \frac{E_a}{RT} \quad \textbf{(Eq. S6)}$$

We also estimated the entropy and enthalpy of activation. By using the Eyring equation in the form

$$k_d = \frac{k_B T}{h} \exp\left(\frac{\Delta S^\ddagger}{R} - \frac{\Delta H^\ddagger}{RT}\right) \quad \textbf{(Eq. S7)}$$

Which is convenient to fit with a linear function as follows:

$$R \ln\left(\frac{k_d \cdot h}{k_B \cdot T}\right) = -\Delta H^\ddagger \left(\frac{1}{T}\right) + \Delta S^\ddagger \quad \textbf{(Eq. S8)}$$

Note that here R is gas constant.

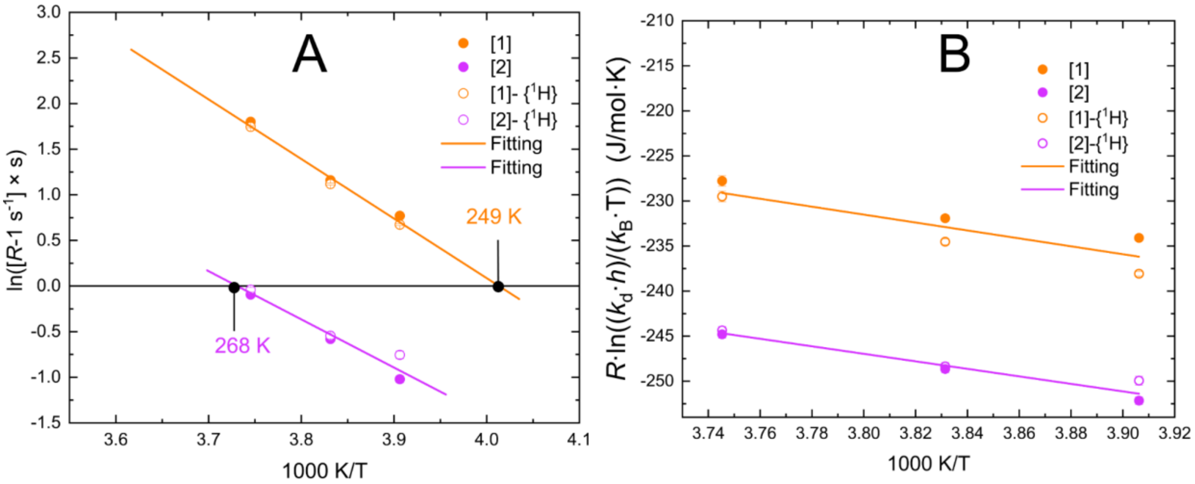

Figure S11: Dissociation rate in function of temperature. Dissociation rates of [1] and [2] were plotted using Arrhenius's equation (A) and using Eyring's equation (B). The relaxation rate for hydrogen is estimated to be 1 s^{-1}. The dissociation rate reaches 1 s^{-1} for [1] at 249 K and for [2] at 268 K. The estimated energy parameters for complex [1] and [2] are in the table below:

	E_a (kJ/mol)	ln(A)	$\Delta S^\ddagger$(J/(mol · K))	$\Delta H^\ddagger$ (kJ/mol)
[1], [1]-{^{1}H}	0.78 ± 0.05	26.21 ± 1.43	-64.39 ± 62.23	43.97 ± 16.10
[2], [2]-{^{1}H}	0.63 ± 0.06	19.71 ± 2.02	-88.47 ± 18.8	41.70 ± 4.91

Other hyperpolarized complexes

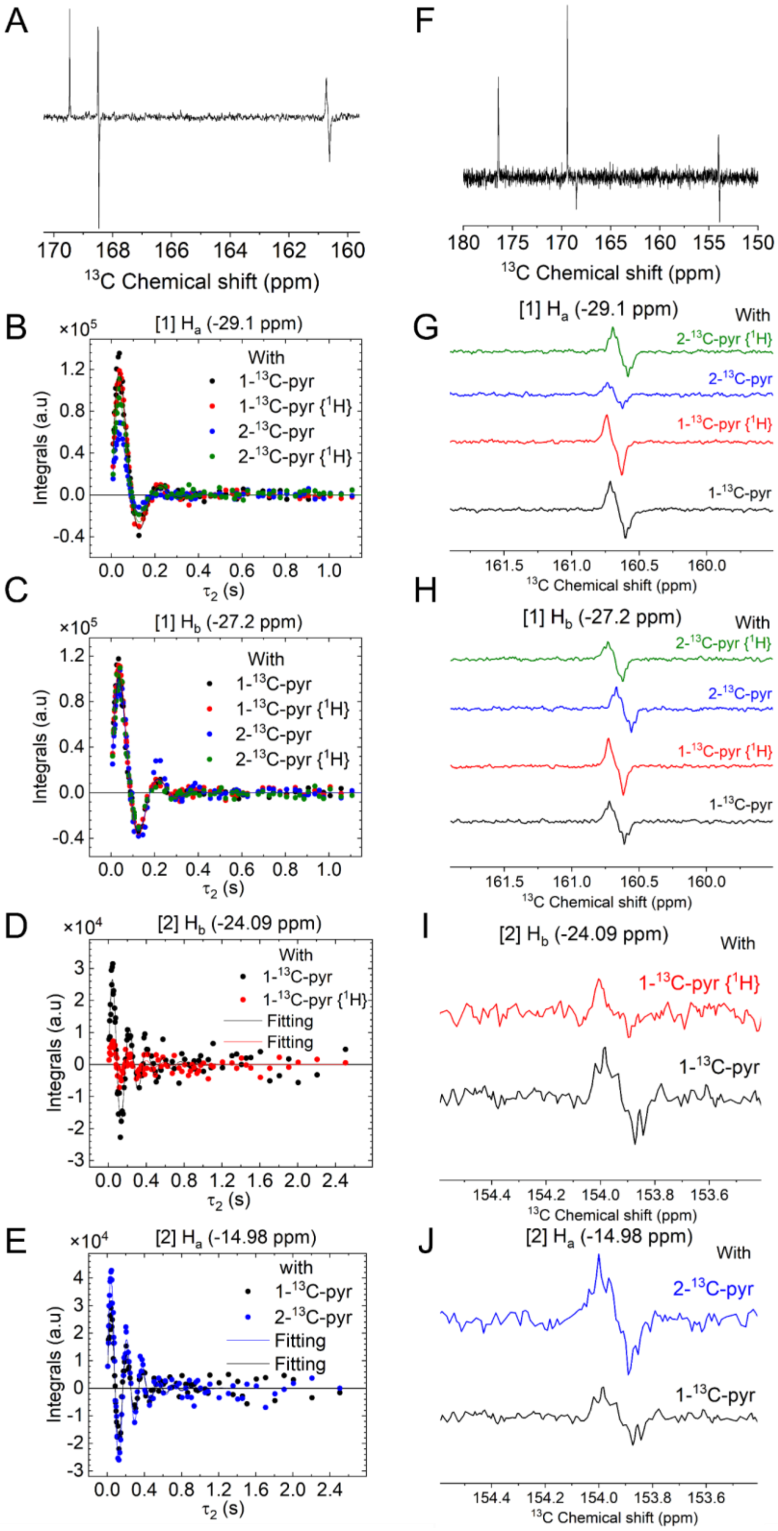

Figure S12: ^{1}H-^{13}C interaction of other hyperpolarized complexes. ^{13}C spectrum of the interaction at 160.64 ppm (A), at 154.10 ppm (F). Fitted integrals of ^{13}C signals of the unknown complex at 160.64 ppm after SEPP-SPINEPT as a function of τ_2, during measurement of [1] for both hydride Ha and Hb (B,C) and of [2] (D,E). Phased spectra were integrated (dots) and fitted with $A \times \sin(2\pi J_{CH}\tau_2) \times \exp(-2\tau_2 R)$ (lines). (G, H, I, J) exemplary phased spectra at a maximum of polarization with signal enhancement. Kinetics measured using 1-^{13}C_pyr (black), 2-^{13}C-pyr (blue). Kinetics measured using 1-^{13}C-pyr and applying {^{1}H} decoupling (red), and 2-^{13}C-pyr and applying {^{1}H} decoupling (green).

During the measurement of interactions for complex [1] using both 1-^{13}C- and 2-^{13}C-pyruvate, an unexpected interaction was observed with ^{13}C with 160.64 ppm resonance. The observed coupling constant was approximately 5.5 Hz and R ~ 6.5 s^{-1}. Similarly, while measuring interactions for complex [2] the interaction with the ^{13}C with a 154.10 ppm resonance was observed. The estimated J coupling constant was around 5.8 Hz and R ~ 2.5 s^{-1}.

Same kinetics were measured with and without applying {^{1}H} decoupling one can notice that when using 1-^{13}C_pyr with decoupling the effect of coupling is eliminated, however it is not the case when using 2-^{13}C-pyr. Therefore, we rule out the hypothesis that this complex contains pyruvate. We could think that this is related to DMSO or methanol.

Simulation of exchange with noise

We tested five parameters of relaxation and dissociation rates $R = 0\ s^{-1}$, $1\ s^{-1}$, $2\ s^{-1}$, $5\ s^{-1}$ and $6.5\ s^{-1}$ for the fixed value of $J = 1$ Hz:

$$\mathrm{Signal}(\tau_2) = \frac{\sin(2\pi J\tau_2)e^{-2R\tau_2}}{\max(\sin(2\pi J\tau_2)e^{-2R\tau_2})} + noise \qquad \textbf{\textit{(Eq. S9)}}$$

Here, we also added noise to the signal to better represent the experiment. This way, we wanted to test the fitting accuracy for different values of rate *R* to find the threshold where we lose accuracy in our estimations. The title of each figure shows the relaxation rate obtained after fitting.

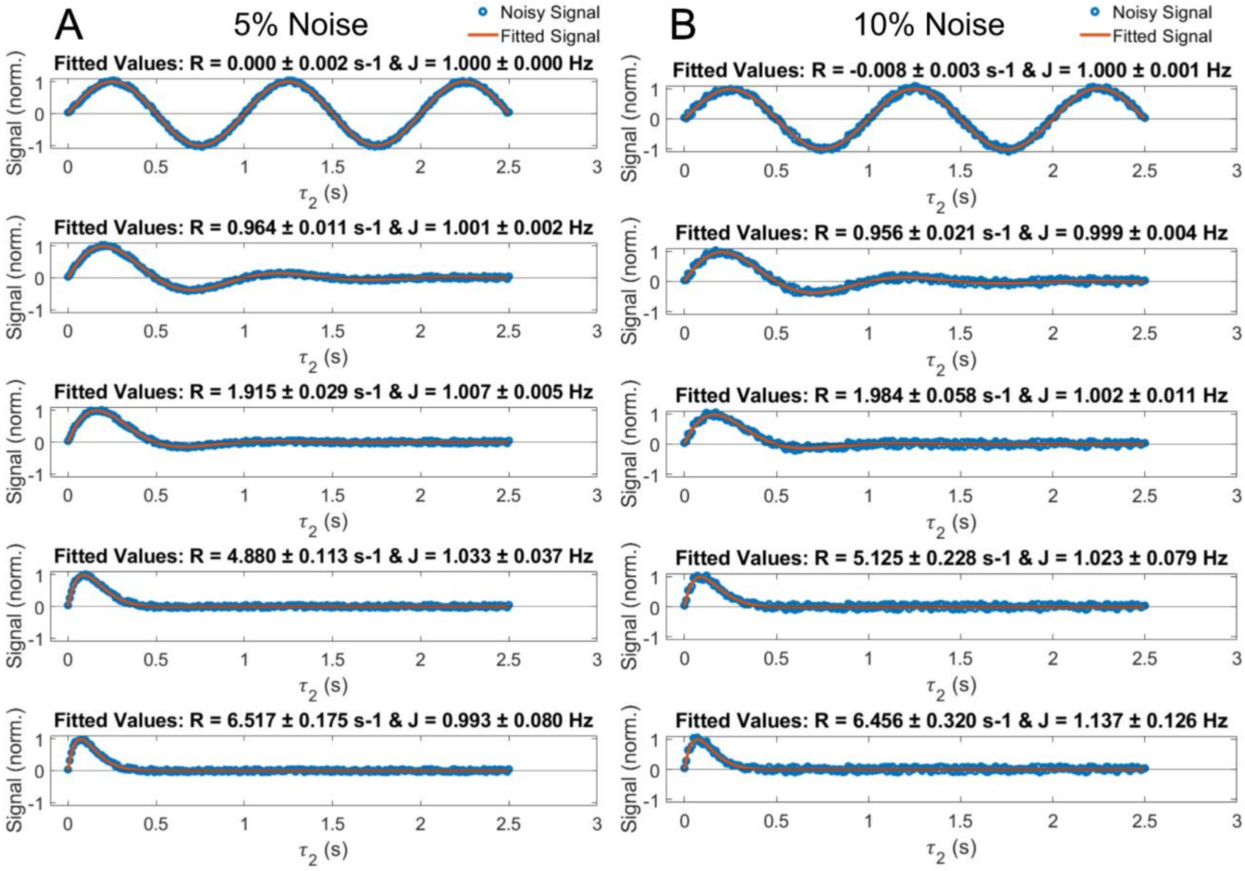

Figure S13: Simulated signal (eq. S9) as a function of relaxation, τ_2 period and noise level. The noise level was 5% (A) and 10% (B). The set parameter *R* was $0\ s^{-1}$, $1\ s^{-1}$, $2\ s^{-1}$, $5\ s^{-1}$ and $6.5\ s^{-1}$, the *J* was 1 Hz, and the result of fit is given in the figure. The estimated J deviates by ~13% when J < R/6.5.

Quantum chemical calculations of the complex structures [1-3] and NMR parameters

Molecular structures optimized at the B3LYP-D4/def2-TZVP level of theory are shown in **Figure S14**. At the optimized structures, NMR chemical shifts calculated at the GIAO-ZORA-TPSSh/def2-TZVPP level of theory and spin-spin coupling constants calculated at the PBE/pcJ-3 level of theory are given in **Table S4**.

In the 2D surface scan for complex [**1**], the H–H and Ir–H_2 distances were varied while keeping Ir–H^a and Ir–H^b distances equal at all times, i.e., two H atoms exhibit symmetrical alignment with respect to the Ir atom. A total of 21 H–H distance values and 18 Ir–H_2 distance values were selected to build a grid of 378 points, each of which corresponded to a constrained geometry optimization at the B3LYP-D4/def2-SVP level of theory. Note that in the obtained potential energy surface (PES) (**Figure 2B**), the energy minimum structure does not exactly coincide with the optimized geometry due to the symmetry restriction on the Ir–H bond lengths and variations in method accuracy. The computed $J_{\text{H-H}}$ and $J_{\text{C-H}}$ when moving from dihydrogen (R_{HH} = 0.8 Å) to the dihydride complex (R_{HH} = 1.9 Å) on the PES (**Figure 2C**) were given in **Table S5.**

Files with the optimised geometries are attached to this manuscript.

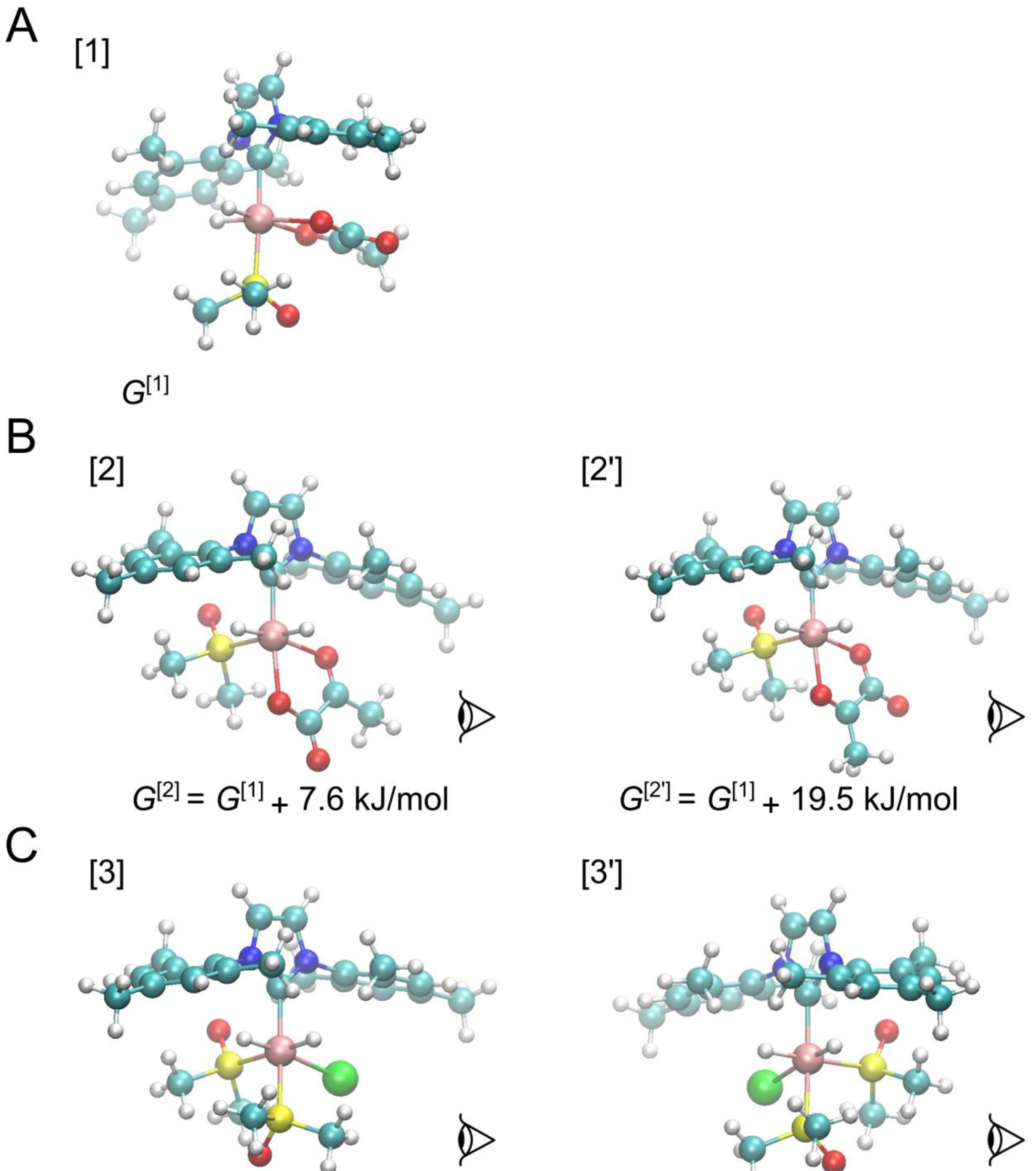

Figure S14: 3D chemical structures for all complexes and their isomers using B3LYP-D4/def2-TZVP. (A) complex [**1**]. (B) complex [**2**] used in our study has 7.6 kJ/mol than [**1**]and [**2'**] an isomer of [**2**] is 19.5 kJ/mol higher in energy than [**1**]. (C) The chiral isomer of [**3**], denoted as [**3'**], is 0.42 kJ/mol lower in energy compared to **[3]**, which is within the error bar of DFT calculations. The eye icons highlight the region of pyruvate reorientation.

Table S4: Computed ^{1}H and ^{13}C NMR chemical shifts (ppm) and coupling constants (Hz) for complexes [**1**] and [**2**] with respect to experimental measurements. The experimental ^{13}C chemical shifts use methanol-*d4* as a reference, and the computational ^{1}H and ^{13}C chemical shifts use TMS as reference.

	[**1**]		[**2**] ([**2'**])	
	Experimental	Calculation	Experimental	Calculation
δ_{Ha} (ppm)	-29.10	-12.1	-14.97	-6.0 (-5.0)
δ_{Hb} (ppm)	-27.20	-9.5	-24.08	-9.9 (-9.4)
δ_{C1} (ppm)	168.54	171.4	165.15	173.2 (170.2)
δ_{C2} (ppm)	206.59	223.8	196.86	218.1 (225.9)
$J_{Ha\text{-}Hb}$ (Hz)	-10.48‡	-0.041	-6.55‡	2.29 (0.912)
$J_{C1\text{-}Ha}$ (Hz)	|0.55 ± 0.02|*<br>|0.53 ± 0.02|†<br>-0.97[27]	0.329	|0.32 ± 0.03|*<br>|0.21 ± 0.04|†	0.338 (0.464)
$J_{C1\text{-}Hb}$ (Hz)	|0.014 ± 0.00|*<br>|0.006 ± 0.00|†<br>0.8[27]	-0.866	|0.25 ± 0.03|*<br>|0.025 ± 0.33|†	0.124 (-1.106)
$J_{C2\text{-}Ha}$ (Hz)	|0.93 ± 0.08|*<br>|0.99 ± 0.016|†<br>-0.5[27]	-1.224	|0.41 ± 0.02|*<br>|0.45 ± 0.02|†	0.809 (0.962)
$J_{C2\text{-}Hb}$ (Hz)	Non-observable*†<br>-0.06[27]	-0.687	|2.69 ± 0.002|*<br>|2.69 ± 0.035|†	-1.477 (-0.883)

* Values measured here using SEPP-SPINEPT as described in the text.
† Values measured here using SEPP-SPINEPT with {^{1}H} decoupling.
‡ Values measured here using SEPP as described in the text.

Table S5: Computed $J_{H\text{-}H}$ and $J_{C\text{-}H}$ at different $R_{H\text{-}H}$ and $R_{Ir\text{-}H2}$ for complex [1].

$R_{H\text{-}H}$ (Å)	$R_{Ir\text{-}H2}$ (Å)	Energy (kcal/mol)	$J_{Ha\text{-}Hb}$ (Hz)	$J_{C1\text{-}Ha}$ (Hz)	$J_{C1\text{-}Hb}$ (Hz)	$J_{C2\text{-}Ha}$ (Hz)	$J_{C2\text{-}Hb}$ (Hz)
0.8	2.587	61.6	291.18	0.00	0.00	0.00	0.00
0.9	1.822	32.2	228.54	-1.55	1.90	0.00	-0.35
1	1.521	18.2	151.85	-1.35	2.27	-0.21	-0.41
1.2	1.418	15.3	69.18	-0.35	-0.48	-0.92	-0.67
1.4	1.372	8.8	33.69	-0.21	-0.67	-0.99	-0.72
1.6	1.316	3.9	15.07	-0.07	-0.75	-1.05	-0.72
1.9	1.212	0.0	3.30	0.20	-0.80	-1.10	-0.65
2.2	1.163	2.6	-1.68	0.45	-0.89	-1.27	-0.63

References

(1) Gelabert, R.; Moreno, M.; Lluch, J. M.; Lledós, A.; Heinekey, D. M. Determination of the Temperature Dependence of the H–D Spin–Spin Coupling Constant and the Isotope Effect on the Proton Chemical Shift for the Compressed Dihydride Complex [Cp*Ir(P–P)H $_2$] $^{2+}$. *J. Am. Chem. Soc.* **2005**, *127* (15), 5632–5640. https://doi.org/10.1021/ja043011r.
