# Supplementary material for: J Coupling Constants of <1 Hz Enable 13C Hyperpolarization of Pyruvate via Reversible Exchange of Parahydrogen
Source: J Phys Chem Lett. 2024 Jan 25;15(5):1195–203. doi: 10.1021/acs.jpclett.3c02980 (PMC10860132; doi:10.1021/acs.jpclett.3c02980)
Supplement: Supplementary file 2 — jz3c02980_si_004.zip [file jz3c02980_si_004.zip › fitting scripts/ReadMe.docx]

# **Fitting Process and DATA**

We employed the SEPP-SPINEPT pulse sequence for each hydride in complexes [1] and [2], and both 1 and 2-^13^C pyruvate, and conducted experiments at three temperatures: 256, 261, and 267 K. Notably, H^b^ (-27.2 ppm) in 2-^13^C complex **[1]** remained unobservable, while H^b^ (-24.09 ppm) in 2-^13^C complex **[2]** exhibited a coupling of 2.69 Hz, well measured using ^1^H PASADENA.

For all observable ^1^H-^13^C interactions, we performed kinetic analysis, fitting the data with the equation A × sin(2π*J*_CH_*τ*_2_)×exp(-2*τ*_2_*R*), where *J*_CH_ represents the *J* coupling between proton and carbon, and *R* denotes the combined relaxation and exchange parameter. Each IrHH proton possesses its distinct *J*_CH_ and exchange constant for each temperature. Notably, IrHH protons within the same complex share the same constant *R*, similar to the one measured with the SEPP (**Table S1**).

To streamline the analysis, we employed a global fitting approach using MATLAB scripts to fit all parameters simultaneously using 9, 10, or 12 kinetics for each complex (depending on the observable kinetics).

For example, when we had 9 data sets (9 kinetics), the fitting was applied across all kinetics with 9 independent amplitudes A_1_ to A_9_, three relaxation-exchange parameters $R$ for each temperature, and three unique *J* coupling interactions.

To use our scripts use the following four scripts:

- Use MATLAB script “Fitting_withoutdc_9SABRE_JCH.m” to fit data for complex [**1**] without decoupling (**Table 1**). Here, the global fit is applied to 9 data sets.
- Use MATLAB script “Fitting_withdc_9SABRE_JCH.m” to fit data for complex [**1**] with decoupling (**Table 2**). Here, the global fit is applied to 9 data sets.
- Use MATLAB script “Fitting_withoutdc_12SABRE_JCH.m” to fit data for complex **[2]** without decoupling (**Table 3**). Here, the global fit is applied to 12 data sets.
- Use MATLAB script “Fitting_withdc_10SABRE_JCH.m” to fit data for complex **[2]** with decoupling (**Table 4**). We did not perform SEPP-SPINEPT kinetics for complex **[2]** with decoupling at 256 and 261 K. Here, the global fit is applied to 10 data sets.

**Table 1**. The sets of parameters used to fit SEPP-SPINEPT kinetics for complex [**1**] without decoupling. See that *J*s are the same for each C-H pair, while *R*s are the same for each T.

| SEPP-SPINEPT | T = 256 K | T = 261 K | T = 267 K |
| --- | --- | --- | --- |
| 1-^13^C-pyruvate, [1]-H^a^ | Set 1 (A_1_, $J_{H^{a}C^{1}}^{\left[ 1 \right]}$, $R_{256}^{\left[ 1 \right]}$  File: | Set 2 (A_2_, $J_{H^{a}C^{1}}^{\left[ 1 \right]}$, $R_{261}^{\left[ 1 \right]}$)  File: | Set 3 (A_3_, $J_{H^{a}C^{1}}^{\left[ 1 \right]}$, $R_{267}^{\left[ 1 \right]}$)  File: |
| 1-^13^C-pyruvate, [1]-H^b^ | Set 4 (A_4_, $J_{H^{b}C^{1}}^{\left[ 1 \right]}$, $R_{256}^{\left[ 1 \right]}$)  File: | Set 5 (A_5_, $J_{H^{b}C^{1}}^{\left[ 1 \right]}$, $R_{261}^{\left[ 1 \right]}$)  File: | Set 6 (A_6_, $J_{H^{b}C^{1}}^{\left[ 1 \right]}$, $R_{267}^{\left[ 1 \right]}$)  File: |
| 2-^13^C-pyruvate, [1]-H^a^ | Set 7 (A_7_, $J_{H^{a}C^{2}}^{\left[ 1 \right]}$, $R_{256}^{\left[ 1 \right]}$)  File: | Set 8 (A_8_, $J_{H^{a}C^{2}}^{\left[ 1 \right]}$, $R_{261}^{\left[ 1 \right]}$)  File: | Set 9 (A_9_, $J_{H^{a}C^{2}}^{\left[ 1 \right]}$, $R_{267}^{\left[ 1 \right]}$)  File: |

**Table 2**. The sets of parameters used to fit SEPP-SPINEPT kinetics for complex [**1**] with decoupling. See that *J*s are the same for each C-H pair, while *R*s are the same for each T.

| SEPP-SPINEPT | T = 256 K | T = 261 K | T = 267 K |
| --- | --- | --- | --- |
| 1-^13^C-pyruvate, [1]-H^a^_dc | Set 1 (A_1_, $J_{H^{a}C^{1}}^{\left[ 1 \right]}$, $R_{256}^{\left[ 1 \right]}$  File:  | Set 2 (A_2_, $J_{H^{a}C^{1}}^{\left[ 1 \right]}$, $R_{261}^{\left[ 1 \right]}$)  File:  | Set 3 (A_3_, $J_{H^{a}C^{1}}^{\left[ 1 \right]}$, $R_{267}^{\left[ 1 \right]}$)  File: |
| 1-^13^C-pyruvate, [1]-H^b^_dc | Set 4 (A_4_, $J_{H^{b}C^{1}}^{\left[ 1 \right]}$, $R_{256}^{\left[ 1 \right]}$)  File:  | Set 5 (A_5_, $J_{H^{b}C^{1}}^{\left[ 1 \right]}$, $R_{261}^{\left[ 1 \right]}$)  File: | Set 6 (A_6_, $J_{H^{b}C^{1}}^{\left[ 1 \right]}$, $R_{267}^{\left[ 1 \right]}$)  File:  |
| 2-^13^C-pyruvate, [1]-H^a^_dc | Set 7 (A_7_, $J_{H^{a}C^{2}}^{\left[ 1 \right]}$, $R_{256}^{\left[ 1 \right]}$)  File: | Set 8 (A_8_, $J_{H^{a}C^{2}}^{\left[ 1 \right]}$, $R_{261}^{\left[ 1 \right]}$)  File:  | Set 9 (A_9_, $J_{H^{a}C^{2}}^{\left[ 1 \right]}$, $R_{267}^{\left[ 1 \right]}$)  File:  |

**Table 3**. The sets of parameters used to fit SEPP-SPINEPT kinetics for complex **[2]** without decoupling. See that *J*s are the same for each C-H pair, while *R*s are the same for each T.

| SEPP-SPINEPT | T = 256 K | T = 261 K | T = 267 K |
| --- | --- | --- | --- |
| 1-^13^C-pyruvate, [2]-H^a^ | Set 1 (A_1_, $J_{H^{a}C^{1}}^{\left[ 2 \right]}$, $R_{256}^{\left[ 2 \right]}$  File:  | Set 2 (A_2_, $J_{H^{a}C^{1}}^{\left[ 2 \right]}$, $R_{261}^{\left[ 2 \right]}$)  File:   | Set 3 (A_3_, $J_{H^{a}C^{1}}^{\left[ 2 \right]}$, $R_{267}^{\left[ 2 \right]}$)  File: |
| 1-^13^C-pyruvate, [2]-H^b^ | Set 4 (A_4_, $J_{H^{b}C^{1}}^{\left[ 2 \right]}$, $R_{256}^{\left[ 2 \right]}$)  File: | Set 5 (A_5_, $J_{H^{b}C^{1}}^{\left[ 2 \right]}$, $R_{261}^{\left[ 2 \right]}$)  File: | Set 6 (A_6_, $J_{H^{b}C^{1}}^{\left[ 2 \right]}$, $R_{267}^{\left[ 2 \right]}$)  File: |
| 2-^13^C-pyruvate, [2]-H^a^ | Set 7 (A_7_, $J_{H^{a}C^{2}}^{\left[ 2 \right]}$, $R_{256}^{\left[ 2 \right]}$)  File: | Set 8 (A_8_, $J_{H^{a}C^{2}}^{\left[ 2 \right]}$, $R_{261}^{\left[ 2 \right]}$)  File: | Set 9 (A_9_, $J_{H^{a}C^{2}}^{\left[ 2 \right]}$, $R_{267}^{\left[ 2 \right]}$)  File: |
| 2-^13^C-pyruvate, [2]-H^b^ | Set 10 (A10, $J_{H^{b}C^{2}}^{\left[ 2 \right]}$, $R_{256}^{\left[ 2 \right]}$)  File: | Set 11 (A_11_, $J_{H^{b}C^{2}}^{\left[ 2 \right]}$, $R_{261}^{\left[ 2 \right]}$)  File: | Set 12 (A_12_, $J_{H^{b}C^{2}}^{\left[ 2 \right]}$, $R_{267}^{\left[ 2 \right]}$)  File: |

**Table 4**. The sets of parameters used to fit SEPP-SPINEPT kinetics for complex **[2]** with decoupling. See that *J*s are the same for each C-H pair, while *R*s are the same for each T.

| SEPP-SPINEPT | T = 256 K | T = 261 K | T = 267 K |
| --- | --- | --- | --- |
| 1-^13^C-pyruvate, [2]-H^a^ | Set 1 (A_1_, $J_{H^{a}C^{1}}^{\left[ 2 \right]}$, $R_{256}^{\left[ 2 \right]}$  File:  | Set 2 (A_2_, $J_{H^{a}C^{1}}^{\left[ 2 \right]}$, $R_{261}^{\left[ 2 \right]}$)  File: | Set 3 (A_3_, $J_{H^{a}C^{1}}^{\left[ 2 \right]}$, $R_{267}^{\left[ 2 \right]}$)  File: |
| 1-^13^C-pyruvate, [2]-H^b^ | Set 4 (A_4_, $J_{H^{b}C^{1}}^{\left[ 2 \right]}$, $R_{256}^{\left[ 2 \right]}$)  File: | Set 5 (A_5_, $J_{H^{b}C^{1}}^{\left[ 2 \right]}$, $R_{261}^{\left[ 2 \right]}$)  File:   | Set 6 (A_6_, $J_{H^{b}C^{1}}^{\left[ 2 \right]}$, $R_{267}^{\left[ 2 \right]}$)  File: |
| 2-^13^C-pyruvate, [2]-H^a^ | Set 7 (A_7_, $J_{H^{a}C^{2}}^{\left[ 2 \right]}$, $R_{256}^{\left[ 2 \right]}$)  File: | Set 8 (A_8_, $J_{H^{a}C^{2}}^{\left[ 2 \right]}$, $R_{261}^{\left[ 2 \right]}$)  File: | Set 9 (A_9_, $J_{H^{a}C^{2}}^{\left[ 2 \right]}$, $R_{267}^{\left[ 2 \right]}$)  File: |
| 2-^13^C-pyruvate, [2]-H^b^ | Not performed | Not performed | Set 10 (A_10_, $J_{H^{b}C^{2}}^{\left[ 2 \right]}$, $R_{267}^{\left[ 2 \right]}$)  File: |
