# Supplementary material for: J Coupling Constants of <1 Hz Enable 13C Hyperpolarization of Pyruvate via Reversible Exchange of Parahydrogen
Source: J Phys Chem Lett. 2024 Jan 25;15(5):1195–203. doi: 10.1021/acs.jpclett.3c02980 (PMC10860132; doi:10.1021/acs.jpclett.3c02980)
Supplement: Supplementary file 4 — jz3c02980_si_007.pdf [file jz3c02980_si_007.pdf]

jz-2023-02980q.R1

Name: Peer Review Information for "J-Coupling Constants Below 1 Hz Enable ¹³C Hyperpolarization of Pyruvate via Reversible Exchange of Parahydrogen"

First Round of Reviewer Comments

Reviewer: 1

Comments to the Author

The manuscript by Assaf et al. describes interesting data and an elegant experimental approach to measuring J-coupling parameters of Ir complexes with 13C-pyruvate ligand. These complexes are very important in the area of spin hyperpolarization using signal amplification by reversible exchange (SABRE). The paper is timely and well described. I believe that this manuscript presents new physical insights, as the precise values of J-coupling constants were under question. Conclusions are adequately supported by the data. However, I think that the following modifications are needed to follow the high standards of JPCL:

1. The manuscript is a bit too long for a letter format. The same goes for the abstract. The authors could consider text shortening, as this may improve the clarity of the presentation.

2. The authors prefer to use “indirect nuclear spin-spin interaction” in the main text and also in the title. I believe that simply “J-coupling constants” is a more clear and precise term in the context of the problems discussed in the manuscript.

3. Limitations of the SEPP-SPINEPT approach could be described, as it seems to be limited to relatively simple, specific ligands such as 13C-labeled pyruvate. It might be difficult to use it for other molecules.

4. Some procedures could be described in more detail. Specifically, fitting procedures such as global fit of SEPP-SPINEPT data, Lorentzian fit from 1H PASADENA, and the estimation of activation energies (also enthalpy and entropy) are described only briefly. It is not clear how the presented error margins for the estimated parameters were determined. Is it some standard way used in MATLAB, or something else? The error margins are narrow, and information about their calculation would be good to see, especially for the spin-spin coupling constants.

5. Main text, abstract, P-2, 39-40: The sentence is hard to read. Is “frequency-selectively pulsed” the correct wording?

6. Main text, P-5,13: I think that >20% was for the deuterated compound.

7. Main text, P-9,7-10: The authors describe that Ha and Hb chemical shifts were known for the studied complexes. Why was [1] reassigned? Was that unknown?

8. Main text, P-10,36: I again wonder if “frequency-selective pulsed insensitive nuclei…” is correct. This sentence is hard to follow.

9. Main text, P-12, Figure 4 caption, 33: It is said that tau1 = 20 ms. Why? 23 ms would be a better choice, though, if I look at the J-coupling constant.

10. Main text, P-13, Figure 5 caption, 32: It is said that tau1 = 36 ms. Similarly to the previous comment, would 38 ms be a better choice?

11. Main text, P-13, 35: SEPP-SPINET -> SEPP-SPINEPT

12. Main text, P-14, 46: Change of R discussed. As I guess from Table 1, this discussion is only for [1]. Is this true?

13. Main text, P-15,30: It is said that the lifetime for [2] is 350 ms. How does it come from the results? Maybe the lifetime is 250 ms? This could be clarified.

14. Main text, P-15,54: It is not described what the source of the IrIMes catalyst is. It is with the deuterated IMes. Where did the authors get this complex?

15. Main text, P-16, 16: “transposition” -> “trans position”. The sentence lacks clarity.

16. Some formulas may have errors or typos. For instance, in SM S-6 top, is it correct that the damping is exp(-R*tau1)? Shouldn't it be exp(-2*R*tau1)? In SM, S-4 top, it is said that Delta = 5 ms, but it must be 10 ms according to the context.

17. Both complexes [1] and [2] have the maximum PASADENA signal at 267 K (Figure S1). Is it a coincidence?

18. Some typos and unclear phrases:

a. SM, S-4: calibrated the duration of the power -> calibrated the power

b. P-2, 43: allowes -> allows

c. P-2,47: ontained -> contained

d. Main text, P-5, 7: reform -> reforms; now ->new

Reviewer: 2

Comments to the Author

Assaf et al. describe an approach to obtain 13C-1H couplings for systems involving pyruvate. Pyruvate has received a lot of attention with respect to signal amplification by reversible exchange (SABRE), with a number of manuscripts published in 2022 / 2023. This is inclusive of the first in-vivo image being obtained using the approach. Understanding the spin-order transfer is therefore key to maximising the signal intensity obtained using the technique. I agree with the authors, that, to date, a number of groups have estimated these couplings to explain results obtained. This manuscript provides a robust treatise of the couplings using SEPP-SPINEPT and are contextualised from a DFT perspective also.

My main criticism of the manuscript is that having obtained these values, the authors do not propose how the spin order transfer could be maximised with the knowledge obtained. Yes, they have clearly demonstrated that some couplings were estimated to be an order of magnitude larger than what they should be, but how do we now, as a community, use this information to further experiments etc? I would like to see this commented on at the end of the manuscript.

I also detail some further comments below. In particular, the abstract and TOC seemed to have been rushed / not evaluated before submission in any great detail. The abstract contained a number of basic errors which I expect should have been readily detected before submission.

I am happy to support publication of this manuscript. I believe that the insight offered will be of upmost interest to those in the SABRE community. The use of hyperpolarisation to obtain some 13C-1H couplings may also be of interest to those working in related fields.

Abstract

Can SABRE be described as a "ground-breaking" technique when DNP has been able to polarise it for greater than a decade?

"that harness up to 5700 folds 13C signal gain" – firstly, fold is not plural here. Secondly does the technique harness this enhancement, or does the application of SEPP-SPINEPT lead to an enhancement of 5700-fold?

Allowes is misspelled

Ontained is misspelled

"finding values" does not come across as scientific

TOC graphic

The value of 0.93 Hz seems rather key, yet it is not cited in the abstract, so therefore can't be important?

What does Arcadians / Space Invaders have to do with this approach?

Introduction

"pretty insensitive" – either NMR is insensitive or it is not – no comparators are given so the word pretty does not aid the reader here.

SOT should be defined (it is in abstract but not in introduction)

Figure 1 is very confusing. It refers to the PASADENA of pyruvate. In my mind this would involve hydrogenation of the substrate. Yet the substrate is unchanged. I see very little relevance of a PASADENA mechanism in a manuscript that is concerned with SABRE. The 4 of methanol-d4 should also be subscript.

R&D

Further to the point above, page 8 of the submitted pdf gives a detailed breakdown of the 1H PASADENA experiment, inclusive of what PASADENA stands for. Could the authors elaborate on what "synthesis" is occurring when this experiment is performed? The pyruvate is not hydrogenated and therefore a new product is not "synthesised". This terminology could be misleading. One assumes that "PASADENA" was utilised here because the polarisation occurs within the magnetic field.

The description of the pHINEPT sequence is not needed in text and figure form.

Author's Response to Peer Review Comments:

Reviewer 1:

Answer:

Dear reviewer, thank you for your time and thoughtful questions and genuine attitude to make the manuscript better. We tried our best to address all your concerns.

Question:

The manuscript is a bit too long for a letter format. The same goes for the abstract. The authors could consider text shortening, as this may improve the clarity of the presentation.

Answer:

Thank you for this point. We shortened the abstract by ¼ and the main text. Indeed, this better conveys our message.

Question:

The authors prefer to use “indirect nuclear spin-spin interaction” in the main text and also in the title. I believe that simply “J-coupling constants” is a more clear and precise term in the context of the problems discussed in the manuscript.

Answer:

We exchanged spins-spin, indirect spin-spin, and J-couplings with the J-couplings for the sake of uniformity.

Question:

Limitations of the SEPP-SPINEPT approach could be described, as it seems to be limited to relatively simple, specific ligands such as 13C-labeled pyruvate. It might be difficult to use it for other molecules.

Answer:

New text after Figure 3 and introduction of the sequence:

This description shows that SEPP-SPINEPT is useful for asymmetric systems as it needs chemically non-equivalent IrHH protons. However, it will not be practical for IrIMes complex with three pyridine ligands, where IrHH are chemically equivalent[14].

Question:

Some procedures could be described in more detail. Specifically, fitting procedures such as the global fit of SEPP-SPINEPT data, Lorentzian fit from 1H PASADENA, and the estimation of activation energies (also enthalpy and entropy) are described only briefly. It is not clear how the presented error margins for the estimated parameters were determined. Is it some standard way used in MATLAB, or something else? The error margins are narrow, and information about their calculation would be good to see, especially for the spin-spin coupling constants.

Answer:

We extended the method section in the main text, added MATLAB scripts, and extended a description on the 1H PASADENA and SEPP-SPINEPT fitting in SM.

New text (main text):

Fitting. ^{1}H PASADENA and SEPP-SPINEPT kinetics were fitted using MATLAB scripts (available in SM). The details on the fitting functions and global fittings are in SM. All error margins for the fitted values are standard deviations estimated using the MATLAB nonlinear regression “*nlinfit*” function.

…

Supporting Information. The following files are available free of charge. Additional ^{1}H and ^{13}C NMR spectra, SEPP and phINEPT polarization transfer sequences, estimation of *J* couplings constants using

^{1}H and ^{1}H{^{13}C} PASADENA spectra (PDF), corresponding raw data with brief description (ZIP), ^{1}H PASADENA and SEPP-SPINEPT fitting matlab scripts (ZIP).
New text (SM, Estimation of J_{CH} from ^{1}H PASADENA spectra):
The fitting was implemented on MATLAB using nonlinear regression function "nlinfit". The fitting script, together with integrals, are available in Supporting Materials. The reported errors are the results of such fitting.

New text (SM, Estimation of J_{CH} for complex [1] and [2] for both 1,2-^{13}C-pyruvate using SEPP-SPINEPT)
To help understand the applied global fitting and parameters, see Table S3. Table S9 illustrates which parameters were used and were the same for some kinetics.
The reported errors are the results of such fits using MATLAB nonlinear regression function "nlinfit". The fitting script, together with integrals, are available in Supporting Materials.

Table S3. The sets of parameters used to fit SEPP-SPINEPT kinetics. See that *J*s are the same for each C-H pair, while *R*s are the same for each T.

SEPP-SPINEPT	T = 256 K	T = 261 K	T = 267 K
1-^{13}C-pyruvate, [1]-H^a	Set 1 (A_1, $J^{[1]}_{H^aC^1}$, $R^{[1]}_{256}$	Set 2 (A_2, $J^{[1]}_{H^aC^1}$, $R^{[1]}_{261}$)	Set 3 (A_3, $J^{[1]}_{H^aC^1}$, $R^{[1]}_{267}$)
1-^{13}C-pyruvate, [1]-H^b	Set 4 (A_4, $J^{[1]}_{H^bC^1}$, $R^{[1]}_{256}$)	Set 5 (A_5, $J^{[1]}_{H^bC^1}$, $R^{[1]}_{261}$)	Set 6 (A_6, $J^{[1]}_{H^bC^1}$, $R^{[1]}_{267}$)
2-^{13}C-pyruvate, [1]-H^a	Set 7 (A_7, $J^{[1]}_{H^aC^2}$, $R^{[1]}_{256}$)	Set 8 (A_8, $J^{[1]}_{H^aC^2}$, $R^{[1]}_{261}$)	Set 9 (A_9, $J^{[1]}_{H^aC^2}$, $R^{[1]}_{267}$)

Question:
Main text, abstract, P-2, 39-40: The sentence is hard to read. Is "frequency-selectively pulsed" the correct wording?

Answer:
We shortened the text for the sake of clarity.

Old text:
Here, we demonstrate an alternative experimental method using frequency-selective excitation of parahydrogen-derived PASADENA polarization followed by frequency-selectively pulsed insensitive nuclei enhanced by polarization transfer (SEPPSPINEPT)

New text:
we demonstrate an experimental method using frequency-selective excitation of parahydrogen-derived polarization SOT sequence (SEPP-SPINEPT)

Question:
Main text, P-4,13: I think that >20% was for the deuterated compound.

Answer:
We corrected this imprecision of this sentence.

New text:

So far, the highest pH_2-based polarization of 1-^{13}C-pyruvate (> 20%) with SABRE was achieved at 50 µT using weak RF irradiation and fully deuterated pyr

Question:
Main text, P-9,7-10: The authors describe that Ha and Hb chemical shifts were known for the studied complexes. Why was [1] reassigned? Was that unknown?

Answer:
Indeed it is not detailed enough described here.

Old text:
Note that the chemical shifts of H^a and H^b protons were assigned before[15], while chemical shifts for the structure **[1]** are tentatively assigned here based on our analysis as discussed below.

New text:
Before, the chemical shifts of the pair of hydride ligands were assigned to the corresponding molecular composition (not specifying which of two hydrides has which chemical shift)[15], while here, we tentatively assign the chemical shifts of protons to the nuclei in the complex as discussed below

Question:
Main text, P-10,36: I again wonder if "frequency-selective pulsed insensitive nuclei…" is correct. This sentence is hard to follow.

Answer:
Indeed, a bit awkward. Although the abbreviation was published like this already, for the sake of readability we change the text as follows.

Old text:
To disentangle all these interactions, we employ frequency-selective excitation of polarization with PASADENA (SEPP) followed by frequency-selective pulsed insensitive nuclei enhanced by polarization transfer (SPINEPT) or an utterly frequency-selective version of SEPP-INEPT[47] (SEPP-SPINEPT, **Figure 3**)[48].

New text:
To disentangle all these interactions, we employ frequency-selective excitation of polarization with PASADENA (SEPP) followed by insensitive nuclei enhanced by polarization transfer (INEPT) with selective pulses (SP); altogether, this is a frequency-selective version of SEPP-INEPT[47] (SEPP-SPINEPT, **Figure 3**)[48].

Question:
Main text, P-12, Figure 4 caption, 33: It is said that tau1 = 20 ms. Why? 23 ms would be a better choice, though, if I look at the J-coupling constant.

And Question:
Main text, P-13, Figure 5 caption, 32: It is said that tau1 = 36 ms. Similarly, to the previous comment, would 38 ms be a better choice?

Answer:
You are right. Still there is a caveat here. Look at the plot here. Due to rapid signal decay the optimum moves to the left. We added few lines in the text to acknowledge this effect.

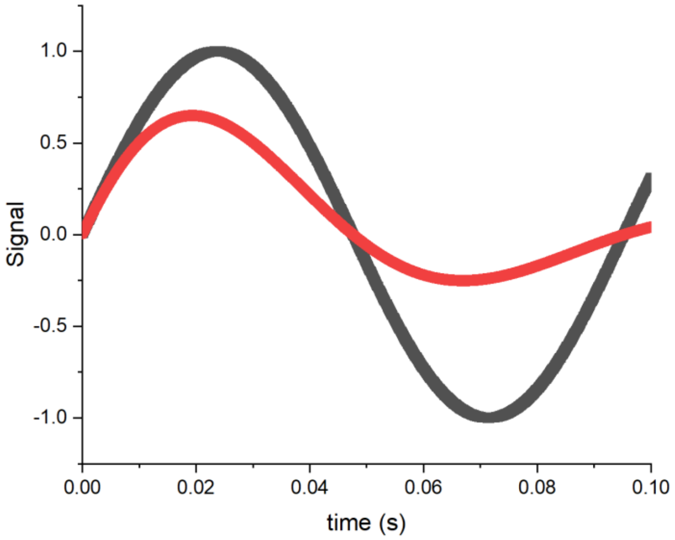

New text, Figure 3, caption:
Due to rapid chemical exchange optimal τ_1 can differ from $1/4J_{IS}$ (examples in SI, Figures S4).

New text, SI, next to Figure S4:

The maximum for complex [1] at 267 K was reached at $\tau_1 \cong 20\ \mathrm{ms} \cong \frac{1}{4J_{\mathrm{HH}}^{[1]}} = 23\ \mathrm{ms}$, for complex [2] at $\tau_1 \cong 38\ \mathrm{ms} \cong \frac{1}{4J_{\mathrm{HH}}^{[2]}} = 38\ \mathrm{ms}$ and for complex [3] at $\tau_1 \cong 29\ \mathrm{ms} \cong \frac{1}{4J_{\mathrm{HH}}^{[3]}} = 41\ \mathrm{ms}$. As one can see, due to signal decay, optimal τ_1 differs from predicted $\frac{1}{4J_{\mathrm{HH}}^{[2]}}$. As one can see, **[3]** has the fastest *R* and hence the largest deviation of experimentally optimal τ_1 from theoretically estimated. On contrast, the **[2]** has the slowest *R* and experimentally optimal τ_1 coincides with the theoretically predicted.

Question:
Main text, P-13, 35: SEPP-SPINET -> SEPP-SPINEPT

Answer:
Thank you. The misspell was corrected.

Question:
Main text, P-14, 46: Change of R discussed. As I guess from Table 1, this discussion is only for [1]. Is this true?

Answer:
Correct, we revised the sentence for clarity.

New text:
Variation of the temperature from 267 to 256 K more than halved the effective relaxation parameter *R* for **[1]** from about 7 s^{-1} to about 3 s^{-1} while for complex [**2**], the reduction was less than 1 s^{-1}, highlighting the contributions from the chemical exchange and differences between dynamics of the complexes

Question:

Main text, P-15,30: It is said that the lifetime for [2] is 350 ms. How does it come from the results? Maybe the lifetime is 250 ms? This could be clarified.

Answer:
We revised few paragraphs on this page. The main changes are highlighted below.

Old text:
Enthalpy and entropy of activation in our conditions were estimated as 42.5 kJ/mol and -63.98 J/(mol·K) for complex [**1**] and 20.52 kJ/mol and -158.51 J/(mol·K) for complex [**2**]; however, lifetimes at the same conditions were slightly different: 76 ms for complex [**1**] and 350 ms for complex [**2**] at 267 K (**Table 1**, and **Figure S11B, SM**).

New text:
Assuming that the effective relaxation of the system during the polarization transfer is about 1 s^{-1} (typical order of magnitude for relaxation time of hydride protons), the lifetimes at e.g. 267 K are 0.16 s for complex [**1**] and 1 s for complex [**2**] at 267 K. Using such relaxation estimates, the values for enthalpy and entropy of activation are 43.4 kJ/mol and -64.39 J/(mol·K) for complex [**1**] and 41.7 kJ/mol and -88.47 J/(mol·K) for complex [**2**] (**Figure S11B, SM**).

Question:
Main text, P-16,54: It is not described what the source of the IrIMes catalyst is. It is with the deuterated IMes. Where did the authors get this complex?

Answer:
The reference was added.

Old text:
Perdeuterated Ir precatalyst [Ir-*d22*] = [IrCl(COD)(IMes-*d22*)] (IMes = 1,3-bis-(2,4,6-trimethylphenyl)-imidazol-2-ylidene, COD = cyclooctadiene)),

New text:
Perdeuterated Ir precatalyst [Ir-d_{22}] = [IrCl(COD)(IMes-d_{22})] was synthesized according to Ref.[50], (IMes = 1,3-bis-(2,4,6-trimethylphenyl)-imidazol-2-ylidene,

Question:
Main text, P-16, 16: "transposition" -> "trans position". The sentence lacks clarity.

Answer:
Old text: We assigned the IrHH chemical shifts to the structure of **[1]** such that the largest interaction corresponds to the transposition of the proton and corresponding carbon: in our case, it is H^a (-29.10 ppm) and C^2 (206.59 ppm); however, the measurements of distances using nuclear Overhouser effects would give better structure evaluation.

New text:
We assigned the IrHH chemical shifts to the structure of **[1]** such that H^a (-29.10 ppm) and C^2 (206.59 ppm) with the largest *J*-coupling of 0.93 Hz are in *trans* position; however, this is a tentative assignment, and the measurements of distances using nuclear Overhauser effects would give better structure evaluation.

Question:

Some formulas may have errors or typos. For instance, in SM S-6 top, is it correct that the damping is exp(-R*tau1)? Shouldn't it be exp(-2*R*tau1)?

Answer:
Thank you for highlighting it. You are right. We corrected the text and reported energy values and R parameters and lifetime of the complexes in manuscript and in SI.

Question:
In SM, S-4 top, it is said that Delta = 5 ms, but it must be 10 ms according to the context.

Answer:
You are right. It was a bit confusing and in fact wrong labelled. We changed the scheme to avoid misunderstanding.

New text:
SEPP SOT converts the PASADENA two-spin order into the magnetization of one of them. We set out to find the delays τ and $τ_3$ (**Figure S3A**) of the pulse sequence that provides the highest signal.
We measured the SEPP signal for $τ_3$ of 0, 2.5, 5, and 7.5 ms, the duration of the SPs was Δ= 10 ms, and τ was changing from 0 to 100 ms. In total, 140 experiments were carried out (**Figure S3B**). The maximum signal was achieved at τ = 10 ms and $τ_3 = Δ/2$ = 5 ms. One can explain that adding $τ_3$ allows the proper rephasing of the spins. Below, we will show that SEPP performance is identical when using different durations of RF pulses (**Figure S4**).

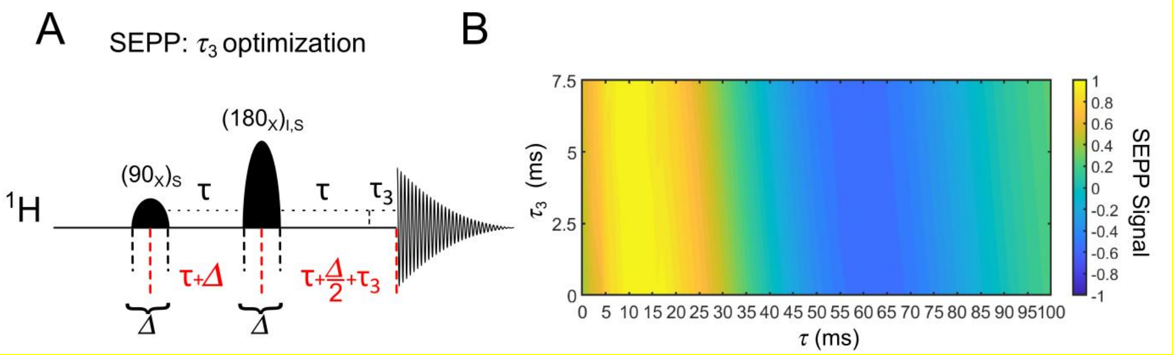

Figure S3. Optimization of SEPP SOT. SEPP sequence (A), the magnetization of H^a (-29.1ppm) of **[1]** after SEPP as a function of τ_3 and τ (B). The highest polarization achieved was at τ = 10 ms, $τ_3$ = 5 ms, and $Δ$ = 10 ms. τ was varied in a step of 1 ms between 0 and 20 ms and in a step of 2 ms between 20 and 100 ms. The highest signal was achieved at τ = 10 ms and $τ_3 = Δ/2$ = 5 ms.

Question:
Both complexes [1] and [2] have the maximum PASADENA signal at 267 K (Figure S1). Is it a coincidence?

Answer:
We do not know. Can be a coincidence but can be an internal dynamic of both complexes which results in such a coincidence. Somebody should study these dependences in more details definitely. However, for us it was not the point. This was just to check is we can indeed polarize protons in whole accessible temperatures regime. The figure serves its purpose.

Question:
Some typos and unclear phrases:
a. SM, S-4: calibrated the duration of the power -> calibrated the power
b. P-2, 43: allowes -> allows

c. P-2,47: ontained -> contained
d. Main text, P-5, 7: reform -> reforms; now ->new

Answer:
Thank you for bringing the misspellings to our attention. All was corrected as requested.

Reviewer 2:
Comments :

Assaf et al. describe an approach to obtain 13C-1H couplings for systems involving pyruvate. Pyruvate has received a lot of attention with respect to signal amplification by reversible exchange (SABRE), with a number of manuscripts published in 2022 / 2023. This is inclusive of the first in-vivo image being obtained using the approach. Understanding the spin-order transfer is therefore key to maximising the signal intensity obtained using the technique. I agree with the authors, that, to date, a number of groups have estimated these couplings to explain results obtained. This manuscript provides a robust treatise of the couplings using SEPP-SPINEPT and are contextualised from a DFT perspective also.

My main criticism of the manuscript is that having obtained these values, the authors do not propose how the spin order transfer could be maximised with the knowledge obtained.

Yes, they have clearly demonstrated that some couplings were estimated to be an order of magnitude larger than what they should be, but how do we now, as a community, use this information to further experiments etc? I would like to see this commented on at the end of the manuscript.

Answer:
We did not study here different SOTs techniques (SHEATH, SLIC etc) as it is beyond the scope of this manuscript. But to address the raised issue without increasing the text much with diverging discussion, we added the following text right at the end of the manuscript.

New text:
Also, the parameters obtained here can be used to numerically optimize SOT to pyruvate.

Question:
I also detail some further comments below. In particular, the abstract and TOC seemed to have been rushed / not evaluated before submission in any great detail. The abstract contained a number of basic errors which I expect should have been readily detected before submission.

Answer:
The abstract and TOC were revised.

I am happy to support publication of this manuscript. I believe that the insight offered will be of upmost interest to those in the SABRE community. The use of hyperpolarisation to obtain some 13C-1H couplings may also be of interest to those working in related fields.

Abstract section:

Question:
Can SABRE be described as a “ground-breaking” technique when DNP has been able to polarise it for greater than a decade?

Answer:
We changed it with:
Signal Amplification By Reversible Exchange (SABRE) has recently emerged as a versatile hyperpolarization technique.

Question:

“that harness up to 5700 folds 13C signal gain” – firstly, fold is not plural here. Secondly does the technique harness this enhancement, or does the application of SEPP-SPINEPT lead to an enhancement of 5700-fold?

Answer:
We changed it with:
We demonstrate an experimental method using frequency-selective excitation of parahydrogen-derived polarization SOT sequence (SEPP-SPINEPT) its application led to up to 5700-fold ^{13}C-signal gain

Question:
Allowes is misspelled
Ontained is misspelled

Answer:
Misspells were corrected.

Question:
“finding values” does not come across as scientific TOC graphic. The value of 0.93 Hz seems rather key, yet it is not cited in the abstract, so therefore can’t be important?
What does Arcadians / Space Invaders have to do with this approach?

Answer:
We had an idea and a debate about it. Thanks to the reviewer, the debate is over. We suggest another graphical abstract which should be cleaner.

Pyruvate SABRE with *J*-couplings < 1Hz

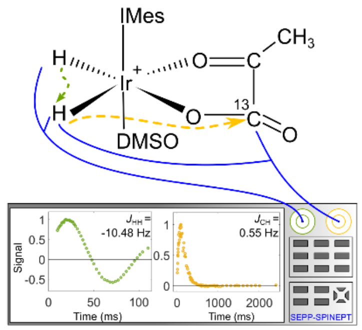

Introduction section:

Question:

“pretty insensitive” – either NMR is insensitive or it is not – no comparators are given so the word pretty does not aid the reader here.

Answer:
The word “pretty” was removed.

Question:
The 4 of methanol-d4 should also be subscript.

Answer:
The 4 of methanol-d4 is now subscript.

Question:
SOT should be defined (it is in abstract but not in introduction).
Answer:
In the letter there is no introduction, to save the space we introduced the sequence when we start using it.
Originally we had a sentence "Here, we propose using spin-selective SOT to measure the 1H-13C J-couplings in complexes directly [1] and [2]." as we also tended to use traditional introduction section. However, we removed this sentence from the second page now for clarity and to follow our logic of bringing things up when necessary.

Question:
Figure 1 is very confusing. It refers to the PASADENA of pyruvate. In my mind this would involve hydrogenation of the substrate. Yet the substrate is unchanged. I see very little relevance of a PASADENA mechanism in a manuscript that is concerned with SABRE.

Answer:
Partially the question is addressed below. We have chemical exchange of H2 with pH2 and as a result IrHH is hyperpolarized and has all characteristics of PASADENA. The only difference from original PASADENA reference is that it binds not to CC bond, but it is not part of definition.
Plus it is easy then to the reader understand why we used SEPP (which according to abbreviation works with PASADENA).
In the figure we did not say hyperpolarized pyruvate but with Pyr. The caption is unambiguous: "(B) ^{1}H (black) and ^{1}H{^{13}C} (red) NMR PASADENA spectra showing polarized IrHH protons of [1-3] formed by supplying 8.75 bar pH_2 to the Ir-catalyst precursor with either 1-^{13}C-pyr (B, top) or 2-^{13}C-pyr (B, bottom) in methanol-d_4 at 267 K."
To reduce confusion, we changed the legends from "1H PASADENA" to "1H PASADENA of IrHH".
As you can see, the term is too convenient not to use.

Results and Discussion Section:

Question:
Further to the point above, page 8 of the submitted pdf gives a detailed breakdown of the 1H PASADENA experiment, inclusive of what PASADENA stands for. Could the authors elaborate on what "synthesis" is occurring when this experiment is performed? The pyruvate is not hydrogenated and therefore a new product is not "synthesised". This terminology could be misleading. One assumes that "PASADENA" was utilised here because the polarisation occurs within the magnetic field.

Answer:
You are right. Pyruvate was not hydrogenated. However, there is chemical exchange and we prepare hyperpolarized short lived Ir-complex (which is synthesised). In addition to this, the term is too much useful and lives for long time without its acronym. We hope that the following corrections will help to avoid ambiguity.

Old text:
To measure these interactions experimentally, one can analyse the ^{1}H-^{13}C *J*-couplings by measuring ^{1}H PASADENA (parahydrogen and synthesis allow dramatically enhanced nuclear alignment)[39] NMR spectra of solutions containing the activated IrIMes catalyst with sodium 1- or 2-^{13}C-pyruvate (1-^{13}C-pyr or 2-^{13}C-pyr), and DMSO-d_6 in methanol-d_4. Three pairs of antiphase resonances for the hydride ligands in the Ir-complexes [**1-3**] (**Figure 1**) were studied. Note that [**1**] and [**2**] are regioisomers

where in [**1**], the pyruvate coordinates *trans* to hydride and in [**2**], pyruvate is *trans* to hydride and NHC; complex [**3**] contains no pyruvate.

New text:
To measure these *J*-couplings experimentally, one can analyze the ^{1}H-^{13}C *J*-couplings by measuring ^{1}H NMR spectra of solutions containing the activated IrIMes catalyst with sodium 1-^{13}C-pyr or 2-^{13}C-pyr, and DMSO-d_6 in methanol-d_4 after addition of pH_2 into solution. Upon hydrogen exchange and addition of pH_2 to IrIMes, three pairs of antiphase resonances for the hydride ligands in the Ir-complexes [**1-3**] (**Figure 1**) are observed. Such NMR spectrum and corresponding spin order, is often called PASADENA (parahydrogen and synthesis allow dramatically enhanced nuclear alignment)[39].

Question:
The description of the pHINEPT sequence is not needed in text and figure form.

Answer:
If we discuss phINEPT as in the question, then we shortened the text in this regard. phINEPT Figure S5 together with kinetics we think is useful as it explains the definition of tau1.
If, however, reviewer meant Figure 3 on the main text, we think it is useful there as the sequence itself is not so common and with only one paragraph we introduce method, assumptions and ways of SEPP-SPINEPT fitting.

Old text:
We started with the parahydrogen and insensitive nuclei polarization transfer sequence (phINEPT)[44], which has already been used for SABRE to enhance ^{15}N signals and to measure lifetime and ligand exchange activation energies for Ir-complexes[43,45]. The sequence starts with a 45° ^{1}H excitation pulse, which is followed by an evolution delay before simultaneous 180° refocusing pulses on ^{1}H and ^{13}C. After another free evolution interval and broadband 90° excitation of both protons and carbons, a ^{13}C spectrum can be detected whose appearance can be changed by variation of one of the evolution periods. This approach allows us to probe the ^{1}H-^{13}C interactions.
However, when using the simple phINEPT approach, it becomes apparent that it is virtually impossible to simultaneously measure two weak ^{1}H-^{13}C J-coupling constants (**Figure S5, SM**). This is because the phINEPT sequence consists of only one spin-refocusing block in which all three interactions between H^a and H^b and the ^{13}C of pyruvate are involved. Hence, considering the short lifetime of these complexes, the resulting analysis is ambiguous.

New text:
We started with the pH_2 and insensitive nuclei polarization transfer sequence (phINEPT)[44], which has already been used for SABRE to enhance ^{15}N signals (and ^{13}C here) and to measure lifetime for Ir-complexes[43,45]. This approach theoretically allows one to probe the *J*-couplings. However, when using the simple phINEPT approach, it becomes apparent that it is virtually impossible to simultaneously measure two weak ^{1}H-^{13}C *J*-coupling constants (**Figure S5, SM**).
